# The Optimization Based Dynamic and Cyclic Working Strategies for Rechargeable Wireless Sensor Networks with Multiple Base Stations and Wireless Energy Transfer Devices

**DOI:** 10.3390/s150306270

**Published:** 2015-03-16

**Authors:** Xu Ding, Jianghong Han, Lei Shi

**Affiliations:** School of Computer and Information, Hefei University of Technology, No.193 Tunxi Road, Hefei 230009, China; E-Mails: dixon.ding@aliyun.com (X.D.); thunder10@163.com (L.S.)

**Keywords:** wireless sensor networks, wireless energy transfer, dynamic and cyclic working schemes, Voronoi diagram, energy replenishing procedure, optimization problem

## Abstract

In this paper, the optimal working schemes for wireless sensor networks with multiple base stations and wireless energy transfer devices are proposed. The wireless energy transfer devices also work as data gatherers while charging sensor nodes. The wireless sensor network is firstly divided into sub networks according to the concept of Voronoi diagram. Then, the entire energy replenishing procedure is split into the pre-normal and normal energy replenishing stages. With the objective of maximizing the sojourn time ratio of the wireless energy transfer device, a continuous time optimization problem for the normal energy replenishing cycle is formed according to constraints with which sensor nodes and wireless energy transfer devices should comply. Later on, the continuous time optimization problem is reshaped into a discrete multi-phased optimization problem, which yields the identical optimality. After linearizing it, we obtain a linear programming problem that can be solved efficiently. The working strategies of both sensor nodes and wireless energy transfer devices in the pre-normal replenishing stage are also discussed in this paper. The intensive simulations exhibit the dynamic and cyclic working schemes for the entire energy replenishing procedure. Additionally, a way of eliminating “bottleneck” sensor nodes is also developed in this paper.

## 1. Introduction

Nowadays, wireless sensor networks (WSNs) have been widely used in environment investigations, disaster monitoring and resource management, *etc.*, since they provide us with more comprehensive information platforms and more advanced techniques in order to deal with monitoring tasks. Researches have been conducted carefully with regard to locating targets [1,2], data routing [3,4,5], and MAC (Medium Access Control) layer issues [6,7,8] in wireless sensor networks. Methods of localizing senor nodes have also been well studied in recent years. Chen *et al.* proposed a range-free localizing algorithm to accurately locate regular nodes in WSNs [9]. In their work, a localizing scheme takes advantages of anchor nodes locations and the possible areas of the neighboring and non-neighboring nodes of regular nodes. Compared with existing localizing schemes using anchor nodes information only, the presented approach provides a significant improvement of the localization accuracy. Additionally, the QoS of WSNs is closely related to the continuous maintenance of sensing coverage. Chen *et al.* introduced a hybrid memetic framework for coverage optimization [10]. They proposed a memetic algorithm, as well as a heuristic recursive algorithm, in their paper. The results from real-world experiments using a WSN test-bed show that the approach proposed is able to maximize the sensing coverage while achieving energy efficiency simultaneously. Wireless sensors are also widely used in tracking physiological index of human body. Mukhopadhyay *et al.* pointed out the usage of wearable sensors in monitoring activities of human beings continuously [11]. Together with advances in sensing technologies, wireless communication technologies, and embedded systems, it is also possible to develop smart human body WSNs for the purpose of health and well-being. Since sensor nodes may be deployed in some harsh environments, such as volcano areas and underwater areas, it turns out to be an ordeal for sensor nodes performing long-term operation. In addition to this external reason, the very cause of hindering sensor nodes from long-term operation is their limited energy reserves provided by installed batteries.

In order to overcome the drawbacks stemming from the limited energy supply, Murphy developed an energy-efficient cross layer scheme to prolong the network lifetime [12]. Gandelli *et al.* comprehensively elaborated advances in antenna technologies for conserving energy in large distributed wireless sensor networks for extensive monitoring tasks [13]. Antonio *et al.* developed an innovative mobile platform for heterogeneous sensor networks. In order to maximize the system performances and the network lifespan, they also worked out a hybrid technique, based on evolutionary algorithms [14]. Some other scientists also developed tactics for sensor nodes to harvest energy from the environment in order to make them function perpetually. Shigeta developed a software control method for maximizing the sensing rate of WSNs, which could harvest energy from the ambient RF power [15]. Yang *et al.* proposed a distributed networking architecture for WSNs [16], which could acquire energy from solar power. In their paper, they presented the AutoSP-WSNs (Automatic Solar Powered Wireless Sensor Networks), which were sensor networks of distributed fashion and could achieve the optimal end-to-end network performance. Li considered the energy efficient scheduling problem of WSNs with energy harvesting [17]. After decomposing the formulated Partially Observable Markov Decision Process (POMDP) into a Markov Decision Process (MDP) problem, the optimal scheduling scheme is obtained by solving the MDP problem.

In addition to adopting energy harvesting techniques, some other scientists turned to mobile sensor nodes or mobile data collectors for help in order to prolong the lifetime of WSNs. Sugihara provided an optimal speed control mechanism [18] for mobile data gatherer with respect to the data transmit latency. Zhao proposed an optimization based distributed algorithm for mobile data gathering [19]. The problem is formulated as a network utility maximization problem complying with constraints regarding to fixed network lifetime. Zhao adopted the Space-Division Multiple Access (SDMA) technique together with the mobility of data collector [20]. In his paper, a region-division and tour-planning algorithm, which could balance the data gathering in different regions, was proposed.

In 2007, Kurs *et al.* published a breaking-through paper [21] in Science that pointed out the feasibility of transferring energy wirelessly through coupled magnetic resonance between the primary and the secondary coils. The energy-transferring manner proposed by Kurs, which takes advantage of the same resonance frequency of both coils, is nonradioactive. Recent years, the wireless energy transfer technique obtains more and more attention. Zhong *et al.* proposed a methodology for making a three-coil wireless energy transfer system more efficient than a two-coil counterpart [22]. Tang *et al.* analyzed the power losses for unsegmented and segmented energy coupling coils for wireless energy transfer [23]. Salas *et al.* applied the wireless energy transfer technique in structural health monitoring [24]. Thoen *et al.* design a coupled wireless power system for mobile data receiver [25]. Li also published a paper regarding the wireless energy transfer technique used in electric vehicles. The power of transmission for electric vehicles could be up to several kilowatts from a relatively long distance [26]. RamRakhyani *et al.* proposed a multiple coils wireless energy transfer system to control the currents selectively in the external and implant coils [27]. The misalignment between the primary and the secondary coils may cause a drop in the energy transfer efficiency. When monitoring rat behaviors, Laskovski *et al.* mounted sensor nodes, which can receive energy from the installed secondary coils, on freely moving rats. He found that the misalignment between the primary and the secondary coils caused by the moving of ratswould lead to insufficient energy reception of sensors [28]. In dealing with this problem, Kilinc *et al.* developed an implanted device for mice with coil tracking system to move the primary coil in order to keep alignment between coils [29]. Russell *et al.* demonstrated a system with 24 overlapping primary coils and ferrite secondary coils [30]. This system could deliver energy to arbitrarily oriented secondary coils. Badr *et al.* also provided a novel configuration of the secondary employing ferrite rods placed at specific locations and orientations with coils to cancel out the misalignment effects [31]. Additionally, many experiments have been conducted to reveal the effects of human presence on wireless energy transfer devices. Chen *et al.* studied the human exposure effects on wireless energy transfer systems working at 0.1–10 MHz. Based on the obtained results, they concluded that the optimal operation frequency of wireless energy transfer systems with human presence should be configured at between 1 and 2.5 MHz [32]. Hong *et al.* studied the specific absorption rate (SAR) of a human exposure to wireless energy transfer systems operating at 1.8 MHz. The model of SAR of a Korean human body in relation to different positions and orientations, as well as the efficiency of the wireless energy transfer facilities, is evaluated [33]. Park *et al.* presented a paper evaluating the transmission efficiency into the inside of a human body with both open-type and close-type wireless energy transfer systems [34]. Oruganti *et al.* found that the human presence will cause a drop in energy transfer efficiency. Based on this phenomenon, they developed a touch/proximity/hover sensing system using such property of wireless energy transfer [35]. They also developed a wireless energy transfer system for power and/or high-data rate transmission using sheet-like waveguides [36]. This system is still capable while transferring energy and/or data through thick metal walls.

In this paper, we find that the wireless energy transfer technique proposed by Kurs is a promising way of replenishing sensor nodes since (1) the energy transferring procedure is not vulnerable to climate changes, and can be performed from a long distance; (2) the energy can be transferred without the line of sight, and it can still be delivered successfully even if there are obstacles between the transferor and the transferee; (3) the energy transferred is clean, which means it will not affect the sending and receiving data procedures in WSNs. Therefore, the wireless energy transfer technique, indeed, sheds light on the promising future of WSNs. Inspired by this novel technique, Xie proposed working schemes for WSNs with wireless energy transfer devices [37]. The scheme developed by Xie is also feasible when multiple sensor nodes are charged by one replenishing device at the same time. However, the model they used in that paper is static, which means that the variables in that model are not functions over time.

Inspired by the device invented by Khripkov [38], we could combine the wireless energy transfer and the mobile data collecting together. The aim of this paper is to provide dynamic and cyclic working schemes for both sensor nodes and wireless energy transfer devices based on a series of optimization problems. In order to achieve this goal, we develop a dynamic network model with variables, which are continuous functions over time. With the help of this dynamic model, we can deduce the constraints that sensor nodes and wireless energy transfer devices should comply with at arbitrary time instance *t*. The optimization problem is firstly established from the dynamic model, and then reshaped into a linear optimization problem, which will yield the identical optimality. The rest of this paper is organized as follows: in Section 2, the working scenario and the preliminary modeling of the prime optimization problem is given. In Section 3, by analyzing the working mechanism of the wireless energy transfer device, the optimization problem established in Section 2 is then transformed into a discrete multi-phased optimization problem. In Section 4, two necessary conditions for the optimality of the optimization problem are proposed. Additionally, the linearization of the discrete optimization problem is carried out in the latter half of this section. In Section 5, issues relating to the pre-normal replenishing stage are studied comprehensively. In Section 6, intensive simulations and numerical analysis are performed. Section 7 concludes this paper.

The main contributions of this paper can be summarized as follows: firstly, we discuss a WSN of multiple base stations in a divide-and-conquer fashion due to the concept of Voronoi diagram. Secondly, the entire energy replenishing procedure is split into the pre-normal and normal replenishing stages. We, then, form a continuous time optimization problem for the normal replenishing cycles in the normal replenishing stage. Later on, the continuous time problem is reformulated into a discrete multi-phased optimization problem with the same optimality by exploiting the working strategies of wireless energy transfer device. In addition, the working schemes for sensor nodes and the wireless energy transfer devices are also obtained for the pre-normal replenishing stage. The intensive simulations exhibit the dynamic and cyclic natures of the working schemes proposed in this paper. In addition to this, a way to eliminate “bottleneck” nodes is also presented in this paper.

## 2. The Description and the Preliminary Modeling of the Problem

### 2.1. Problem Description

In this section, we mainly deal with the modeling issues of the problem, which are mentioned in previous parts of this paper. The goal of modeling this problem, as mentioned in former sections, is to retrieve the dynamic and periodic working strategies for both wireless sensor nodes and wireless energy transfer devices. Before getting fully involved into the discussion of the modeling issues, it would be better to introduce the duties engaged by different components of wireless sensor networks, e.g., sensor nodes, base stations, and wireless energy transfer devices. In the meantime, from the aspect of intelligibility of this article, we table all the symbols used in this paper, which are followed by their corresponding definitions. Please refer to Table 1 for details.

**Table 1:** The symbols used in the paper.

Symbols	Definitions
*D*	The area in which a wireless sensor network is deployed
*S*	The service station
${𝒩}_{B}$	The set of all base stations
$B_{i}$	One base station
$N_{B}$	The number of all sensor stations
${𝒩}_{s}$	The set of all sensor nodes
$s_{i}$	One sensor node
${𝒩}_{W}$	The set of all wireless energy transfer devices
$W_{i}$	One wireless energy transfer device
|⋅|	The cardinality of a set
$SN_{k}$	One sub network
${𝒩}_{k}$	The set of sensor nodes, base station and wireless energy transfer device of $SN_{k}$
$E_{max}$	The initial battery energy of a wireless sensor node
$E_{min}$	The minimum energy required to keep sensor nodes functioning properly
$E_{max}^{i}$	The maximum value of the i-th sensor node in normal replenishing cycles
$E_{min}^{i}$	The minimum value of the i-th sensor node in normal replenishing cycles
P	The path along which the wireless energy transfer device travels
$\tau_{i}$	The time duration spent on charging the i-th sensor node along the travelling path P
$\tau_{S}$	The sojourn time
$R_{i}$	The data generating rate of the i-th sensor node
$R_{ij}(t)$	The sending data rate from the i-th sensor node to the j-th sensor node at time t
$R_{ki}(t)$	The receiving data rate of the i-th sensor node from the k-th sensor node at time t
$R_{iB_{l}}(t)$	The sending data rate from the i-th sensor node to the base station $B_{l}$ sensor node at time t
$R_{iW_{l}}(t)$	The sending data rate from the i-th sensor node to the wireless energy transfer device $W_{l}$ sensor node at time t
$R_{ij}[m]$	The sending data rate from the i-th sensor node to the j-th sensor node in phase m
$R_{ki}[m]$	The receiving data rate of the i-th sensor node from the k-th sensor node in phase m
$R_{iB_{l}}[m]$	The sending data rate from the i-th sensor node to the base station $B_{l}$ sensor node in phase m
$R_{iW_{l}}[m]$	The sending data rate from the i-th sensor node to the wireless energy transfer device $W_{l}$ sensor node in phase m
$I_{ij},I_{iB_{l}}\text{ and }I_{iW_{l}}$	Indicator functions
$e_{i}(t)$	The remaining energy of the i-th sensor node at time t
$p_{i}(t)$	The power of i-th sensor node at time t
$p_{i}[m]$	The power of i-th sensor node in phase m
$C_{ij},C_{ij},C_{ij}\text{ and }\rho$	The power factors
U	The replenishing power
V	The travelling velocity of wireless energy transfer devices
τ	The length of the normal replenishing cycle
$\tau_{P}$	The time duration spent on travelling along P
$D_{P}$	The total length of the travelling path P
$\pi_{0}$	The service station
$\pi_{i}$	The i-th sensor node along the travelling path
$D_{\pi_{i}\pi_{i+1}}$	The distance between two successive points along the travelling path
$t_{i}$	The time instance at which the wireless energy transfer device arrives at the i-th sensor node in normal replenishing cycles
𝒨	The index set of phases
$T_{i}$	The time interval relating to $\tau_{i}$
$O_{i}(\phi )$	The objective value yield by the solution ϕ to the optimization problem, OPT-i
$G_{m}(V,E)$	The data routing scheme in phase *m* in the normal replenishing cycles
$\tau^{pn}$	The length of the pre-normal replenishing stage
$t_{i}^{pn}$	The time at which the wireless energy transfer device arrorives at the i-th sensor node in pre-normal replenishing stage
$\left(G_{m}^{pn}(V,E),\tau_{m}^{pn}\right)$	The data routing scheme in phase m in the pre-normal replenishing stage
$E_{i}$	The initial energy of the i-th sensor node at the beginning of each normal replenishing cycle
$U_{i}^{pn}$	The replenishing power used be wireless energy transfer devices in the pre-normal replenishing stage when charging the i-th sensor node

**Figure 1:**
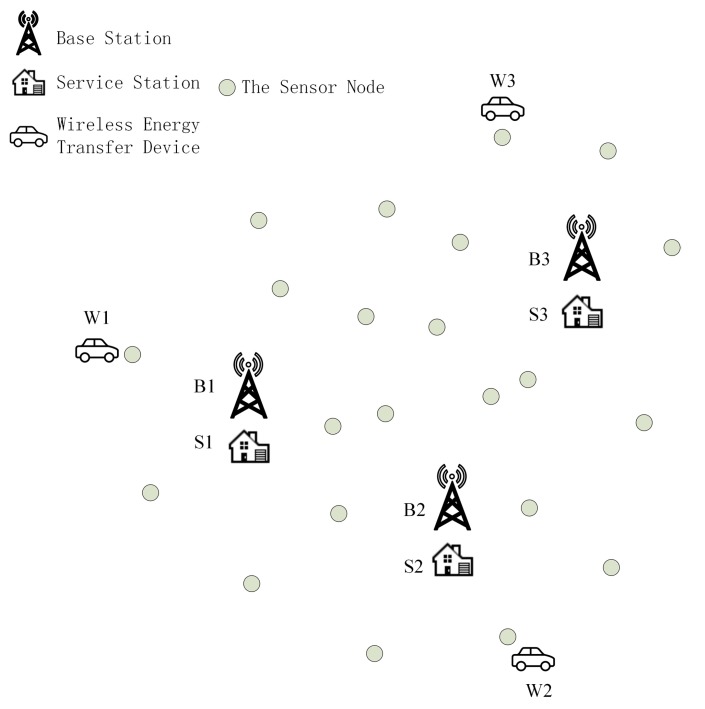
The wireless sensor network with multiple base stations and wireless energy transfer devices.

The wireless sensor network discussed in the scope of this paper is supposed to be located in a certain two-dimensional area, shown in Figure 1, and the area is denoted as D. This wireless sensor network is equipped with several base stations and couple of sensor nodes. The set of all base stations is denoted as $N_{B}=\left\{B_{1},B_{2},B_{3},...,B_{N_{B}}\right\}$. The symbol $B_{i}$ represents a single base station, and $N_{B}$ is the number of base stations. Similarly, the set of all sensor nodes is embodied with $N_{s}=\{s_{1},s_{2},s_{3},...,s_{N_{s}}\}$, where $s_{i}$ denotes a sensor node, and $N_{s}$ is the number of sensor nodes in this WSN. There are $N_{W}$ wireless energy transfer devices awaiting workload assignment. The set of all wireless energy transfer devices is $N_{W}\text{=}\left\{W_{1},W_{2},W_{3},...,W_{N_{W}}\right\}$, where $W_{i}$ denotes one of them. Each sensor node participates in environment data gathering after deployment. Additionally, certain amount of sensor nodes also play the role of routing nodes which are responsible for rallying the acquired date back to one base station and/or one wireless energy transfer device. Base stations are working as data sinks in which the tasks are to store, process, and/or exchange the received data with other data centers, *etc*. The wireless energy transfer devices discussed in this paper have to perform two main functions. On the one hand, the device roams around the area D, and charges sensor nodes wirelessly in case that their remaining battery energy falls below certain level. On the other hand, it plays the role of a data mule while replenishing battery energy of sensor nodes, simultaneously. In order to ensure that sensor nodes will not malfunction due to the insufficient energy supply, the working schemes of both sensor nodes and wireless energy transfer devices should be designed and conducted carefully, which is the main problem to be dealt with in this paper.

### 2.2. Subnet Partition Using Voronoi Diagram

**Figure 2:**
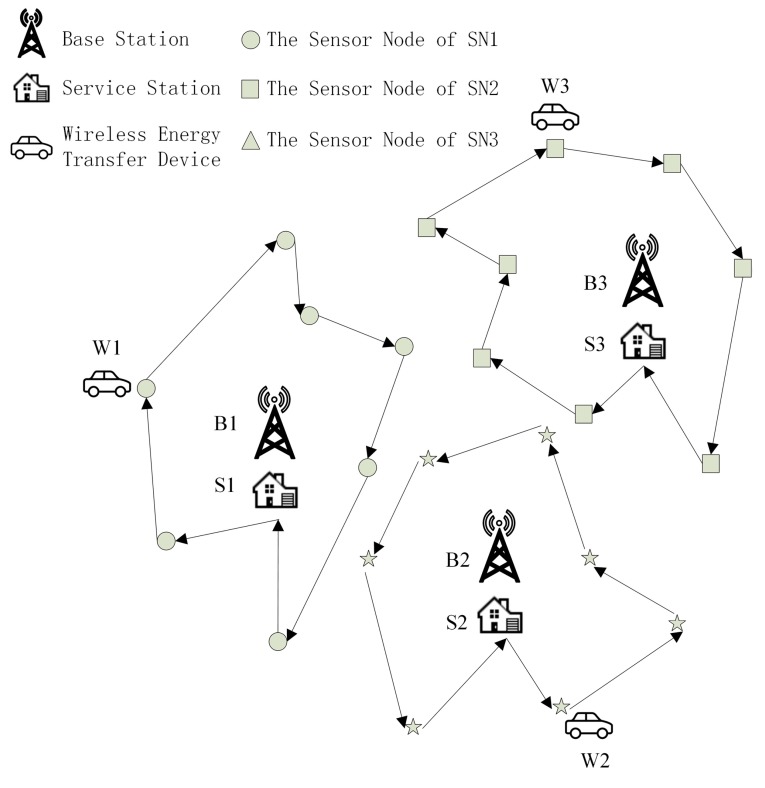
The wireless sensor network divided into 3 sub networks.

Intuitively, discussions of the problem stemming from WSNs with multiple base stations could be carried out in a so-called divide-and-conquer fashion. In this paper, the whole WSN is firstly divided into a number of sub networks, which is shown in Figure 2. Each sensor node is within the “reign” of only one base station, that is, the data gathered by a sensor node will be directed to its own base station. Then, the modeling of the whole WSN could be casted as modeling each sub network one after the other.

The subnet partition scheme adopted here is based on the Voronoi diagram. The definition of Voronoi diagram is described as follow.

**Definition 1.** *(Voronoi diagram) Space* X(X≠∅) *is endowed with a binary operation, denoted as* d:X×X↦ℝ*, where* ℝ *is the set of all real numbers. For arbitrary* x,y∈X*, if the binary operation* d *satisfies:*

(I) d(x,y)≥0*;*
(II) d(x,y)=d(y,x)*;*
(III) d(x,y)≤d(x,z)+d(z,y)

Then d defines a “distance” on X. Suppose that K is an index set, and $P_{k}(k\in K)$ is a tuple consisting of several nonempty sets in X. If the set of elements in X, denoted as $R_{k}$ satisfies: $d(x,P_{k})\leq d(x,P_{j}),\forall x\in R_{k},j\neq k$, where $d(x,P_{k})=\inf_{y\in P_{k}}\{d(x,y)\}$, then $R_{k}$ is a Voronoi region of the set X. Voronoi regions generated by all $P_{k}(k\in K)$ form a Voronoi diagram of the set X.

**Figure 3:**
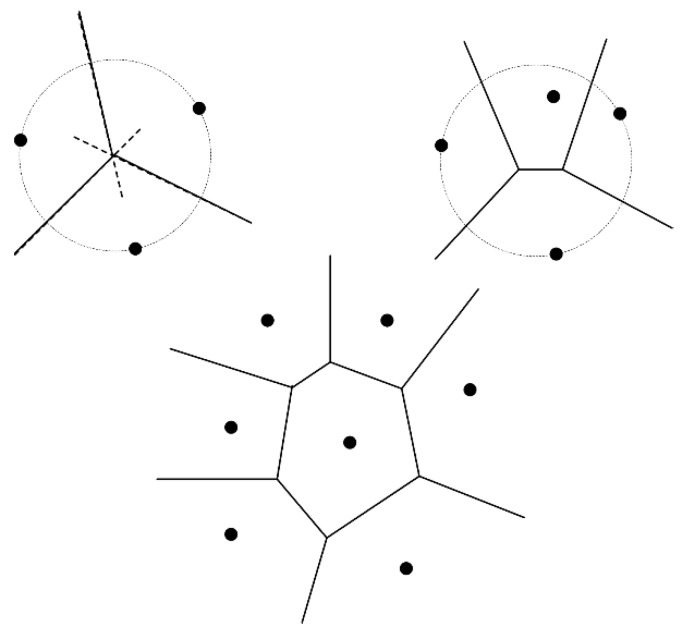
Examples of some Voronoi regions.

The space X , specifically in this paper, is the area D (assuming that the location information of sensor nodes and base stations, which can be acquired by several means, such as GPS, is revealed to us beforehand). The index set K is equal to $\left\{0,1,...,\left|{𝒩}_{B}\right|-1\right\}$, where the operation |⋅| gives the cardinality of a set. Meanwhile, let the tuple $P_{k}(k\in K)$ equal to the coordinates of $B_{k}$. The binary operation d defined on space X is according to the Euclidean distance. Consequently, the Voronoi region $R_{k}$ of $P_{k}\;(k\in K)$ is a polygon of at most $\left|N_{B}\right|-1$ edges, which is shown in Figure 3. Furthermore, there are at most $\left|N_{B}\right|$ Voronoi regions embedded in D. Sensor nodes and base station $B_{k}$ within the same Voronoi region form one sub wireless sensor network, denoted as $SN_{k}$. The set of all sensor nodes in $SN_{k}$ is denoted as $N_{k}$.

### 2.3. The Modeling of Normal Replenishing Cycles

Generally speaking, as we put in previous paragraphs, there are $\left|N_{B}\right|$ subnets in the entire WSN. For subnet $SN_{k}$, there are one base station, one wireless-energy transfer device, and $\left|N_{k}\right|$ sensor nodes. Each sensor node samples the environment information every fixed time interval, which produces the data at the rate of $R_{i}$ $\left(1\leq i\leq N_{k}\right)$ bits/s. Every sensor node is equipped with one fully charged wirelessly rechargeable battery, and the initial energy of it is $E_{max}$. When the remaining energy of the battery falls below certain level, known as $E_{min}$, the sensor node cannot fulfill its functions properly. The wireless energy transfer device $W_{k}$ roams within D, and charges sensor nodes in order to keep them alive. While charging, the replenishing device can also retrieve data directly from the sensor node being charged concurrently. The time interval that the device spends on charging sensor node $s_{i}$ $\left(s_{i}\in SN_{c}\right)$ is denoted as $\tau_{i}$. After charging all sensor nodes, it returns to service station S and stays there for the time period of $\tau_{S}$.

**Figure 4:**
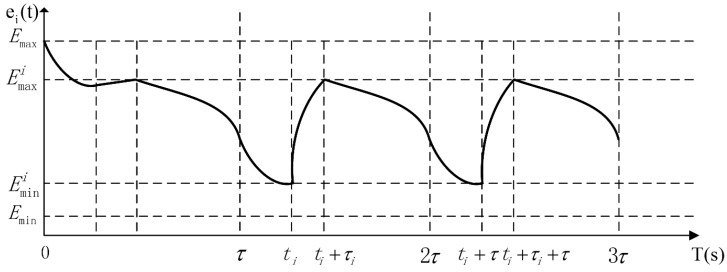
The energy-time curve of a sensor node in pre-normal replenishing stage, the first and the second normal replenishing cycles.

The total replenishing procedure could be categorized into two distinct stages, *i.e.*, the pre-normal replenishing stage and the normal replenishing stage. The pre-normal replenishing stage corresponds to the first replenishing duty executed by the wireless energy transfer device, and the normal one corresponds to the rest replenishing duties. Furthermore, the normal replenishing stage is composed of a series of normal replenishing cycles of time duration τ. The pre-normal replenishing stage will be carefully examined in Section 5. As is shown in later sections, the normal replenishing cycles are periodic extensions of the first normal replenishing cycle. Therefore, we will take the first normal replenishing cycle as an example while modeling the WSN. The energy-time curve of the sensor node $s_{i}$ is depicted in Figure 4, revealing the pre-normal replenishing stage, the first, and the second normal replenishing cycles.

Obviously, the problem we want to attack in this paper involves networking issues together with energy issues of WSNs. Hence, the modeling of WSNs with wireless energy-transfer devices should, of course, exploit both networking and energy properties of each network component. Assume that at the time instance t in the first normal replenishing cycle, the sensor node $s_{i}$ sends data to $s_{j}$, and the sending data rate is $R_{ij}(t)$ bits/s, and $s_{i}$ receives data from $s_{k}$ at the rate of $R_{ki}(t)$ bits/s. Nevertheless, $s_{i}$ may transmit data directly to one base station or one wireless-energy transfer device at the rate of $R_{iB_{l}}(t)$ bits/s or $R_{iW_{l}}(t)$ bits/s, separately. Hence, at $s_{i}$, the following constraint must be satisfied:

(1) $$R_{i}+\sum_{k\in {𝒩}_{s},k\neq i}I_{ki}R_{ki}(t)=\sum_{j\in {𝒩}_{s},j\neq i}I_{ij}R_{ij}(t)+\sum_{B_{l}\in {𝒩}_{B}}I_{iB_{l}}R_{iB_{l}}(t)+\sum_{W_{l}\in {𝒩}_{W}}I_{iW_{l}}R_{iW_{l}}(t)\;(t\in \left[\tau ,2\tau \right))$$

where the $I_{ij}$, $I_{iB_{l}}$ and $I_{iW_{l}}$ are indicator functions, *i.e.*, for instance:

$$I_{ij}=\{\begin{array}{c} 1\text{ if }s_{i}\text{ and }s_{j}\text{ belong to the same sub network}\\ 0\text{ otherwise } \end{array}$$

In order to keep sensor node $s_{i}$ functioning appropriately during the first normal replenishing cycle, the remaining energy at arbitrary time instance t, denoted as $e_{i}(t)$, should satisfy:

(2) $$E_{min}\leq e_{i}(t)\leq E_{max}\;(t\in \left[\tau ,2\tau \right))$$

and the power usage at time instance t, known as $p_{i}(t)$, can be calculated as:

(3) $$\begin{array}{l} p_{i}(t)=\sum_{k\in {𝒩}_{s},k\neq i}\rho I_{ki}R_{ki}(t)+\sum_{j\in {𝒩}_{s},j\neq i}C_{ij}I_{ij}R_{ij}(t)+\sum_{B_{l}\in {𝒩}_{B}}C_{iB_{l}}I_{iB_{l}}R_{iB_{l}}(t)+\sum_{W_{l}\in {𝒩}_{W}}C_{iW_{l}}I_{iW_{l}}R_{iW_{l}}(t)\\ (t\in \left[\tau ,2\tau \right) \end{array}$$

where ρ denotes the power factor associated with receiving per unit data from other sensor nodes, while $C_{ij}$, $C_{iB_{l}}$ and $C_{iW_{l}}$ relate to power factors when transmitting per unit data to other sensors, base stations and wireless energy-transfer devices, respectively.

As shown in Figure 4, the energy consumption of sensor node $s_{i}$ within any normal replenishing cycle should be equivalent to the energy replenished by the wireless energy transfer device during $\tau_{i}$, which is also the requirement of designing cyclic working schemes for both sensor nodes and replenishing devices. Therefore, the following equation should also be met:

(4) $$U\tau_{i}=\int_{t=\tau }^{2\tau }p_{i}(t)dt$$

Equations (1)–(4) are constraints that a sensor node should satisfy. In other words, they are the very constraints that contribute to the development of the cyclic working strategies for sensor nodes. The rest constraints or equations are related to wireless energy transfer devices which will help us regulate the behaviors of them.

On the behalf of the replenishing device, the entire normal replenishing cycle τ can be split into three main parts, *i.e.*,

(5) $$\tau =\sum_{i}\tau_{i}+\tau_{S}+\tau_{P}$$

where $\tau_{P}$ represents the time spent on roaming around the area D, and P is the path taken by a replenishing device.

(6) $$\tau_{P}=\frac{D_{P}}{V}=\frac{\sum_{i=0}^{N_{k}}D_{\pi_{i}\pi_{\left(i+1)\mathrm{mod}(N_{c}+1\right)}}}{V}\;$$

where $D_{p}$ is the total length of the path P, and V is the travelling velocity of a replenishing device. $\pi_{i}(i\neq 0)$ stands for the i-th sensor node along the travelling the path P. $D_{\pi_{i}\pi_{\left(i+1)\mathrm{mod}(N_{c}+1\right)}}$ is the distance between two successive spots along the travelling path.

Our aim, by introducing wireless energy transfer devices into wireless sensor networks, is to keep sensor nodes from malfunctioning caused by the lack of energy reserves. However, paradoxically, we do not want these devices to be so “hardworking”. On the contrary, it is fabulous for these devices staying at service stations as long as possible while keeping all sensor nodes above certain energy level. Therefore, evidentially, we have the following optimization problem OPT-1:

$$\begin{array}{l} \text{OPT-1}\\ \max:\frac{\tau_{S}}{\tau }\\ \text{subject to}:(1)-(6) \end{array}$$

The objective function of this optimization problem stands for our pursuit that the sojourn time of a replenishing device at service station S should be as long as possible. $\frac{\tau_{S}}{\tau }$ is called the sojourn time ratio in this paper. The optimization variables are $R_{ki}(t)$, $R_{ij}(t)$, $R_{iB_{l}}(t)$, $R_{iW_{l}}(t)$, P, $\tau_{i}$, $\tau_{P}$, $\tau_{S}$, $e_{i}(t)$ and all the indicator functions. Before carrying on our discussion on the above optimization problem, we need to state two properties in advance.

**Property 1.** *Assume that in the normal replenishing cycle, the wireless energy transfer device arrives at sensor node* $s_{i}$ *at* $t_{i}$*, and leaves at* $t_{i}+\tau_{i}$ *in the first normal replenishing cycle. Then, the remaining energy of* $s_{i}$ *achieves its maximum at* $t_{i}+\tau_{i}$*. Moreover, it falls to its minimum at* $t_{i}$*. Therefore, the energy constraint Equation (2) is equivalent to:*

(7) $$e_{i}(t_{i})\geq E_{min}$$

(8) $$e_{i}(t_{i}+\tau_{i})\leq E_{max}$$

**Proof.** Please refer to the Appendix for more details.

**Figure 5:**
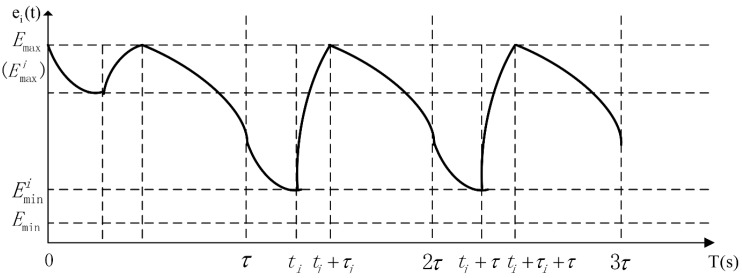
The energy-time curve of a fully charged sensor node.

**Property 2.** *If each sensor node is fully charged, i.e., the remaining energy of* $s_{i}$ *at* $t_{i}+\tau_{i}$ *achieves* $E_{max}$*, the optimality of OPT-1 will remain the same, as shown in* *Figure 5**, which indicates that OPT-1 will yield the same optimal objective value no matter if the sensor nodes are fully charged or not. Therefore, the constraint Equation (8) can be substituted with:*

(9) $$e_{i}(t_{i}+\tau_{i})=E_{max}$$

Consequently, the OPT-1 is then reformulated as:

$$\begin{array}{l} \text{OPT-2}\\ \max:\frac{\tau_{S}}{\tau }\\ \text{subject to}:(1),(3)-(7),(9) \end{array}$$

## 3. Modeling the Normal Replenishing Cycle from a Multi-Phased Aspect

In the previous section, we have deduced the optimization problem of wireless sensor networks with wireless energy transfer devices, *i.e.*, OPT-1. With the help of Property 1 and 2 listed above, OPT-1 is then reformulated into OPT-2. At first examination of this optimization problem, it is hard to find an efficient way to obtain the optimal solution in that the variables are continuous functions of time, and the constraints of OPT-2 contain large amount of product and integral terms. In this section, we will firstly form another optimization problem OPT-3 from a multi-phased aspect. Then, by proving the equal optimality of OPT-3 and OPT-2, our focus on solving OPT-2 would be altered to find an optimal solution to OPT-3.

### 3.1. The Analysis of Working Phases of a Wireless Energy Transfer Device

Generally speaking, within one normal replenishing cycle, the working phases of a wireless energy transfer device fall into two categories. During the first phase, the wireless energy transfer device has no interaction with sensor nodes deployed in area D, *i.e.*, it travels along its path or stays at service station S. During the other phase, it charges each sensor node and collects the data from the sensor node being charged. Furthermore, if the sub network $SN_{c}$ contains $N_{c}$ sensor nodes, the second phase can be further divided into $N_{c}$ sub phases. Therefore, any normal replenishing cycle of sub network $SN_{c}$ can be split into $\left(N_{c}+1\right)$ phases. Let ℳ be the index set of all $\left(N_{c}+1\right)$ phases, *i.e.*, $ℳ=\left\{0,1,2,...,N_{c}\right\}$. Phase 0 corresponds to the time spent on roaming and staying at service station. Phase $i(i=1,2,...,N_{c})$ is related to charging and gathering data from the i-th sensor node along the travelling path.

Let $T_{i}$ represent the time interval associated with the i-th phase. Then, we have:

$$\left\{ \begin{array}{l} T_{i}=\left\{ \begin{array}{l} \left[t_{i},t_{i}+\tau_{i}\right)\;i=1,2,...,N_{c}\\ \left[\tau ,2\tau \right)-\cup_{i=1}^{N_{c}}T_{i}\;i=0\; \end{array}\right.\\ \tau_{0}=\tau -\sum_{i=1}^{N_{c}}\tau_{i} \end{array}\right.$$

where $\tau_{0}$ is the duration of $T_{0}$.

### 3.2. The Discrete Model with Respect to Multiple Phases

In order to get rid of the integral terms contained in the constraint Equation (4) of OPT-2, certain compromise on the flexibility of variables is made in this paper. Obviously, the optimization variables in OPT-2, such as $R_{ki}(t)$, $R_{ij}(t)$, $R_{iB_{l}}(t)$, and $R_{iW_{l}}(t)$ are all continuous over time t, which is the reason for the existence of the integral terms. We want to make above variables less flexible while this optimization problem still yields the identical optimality. The flexibility of variables is compromised by fixing the variables in each phase m∈ℳ, *i.e.*, these variables are constant during each phase, however, values of them may be exposed to change among different phases.

The continuous optimization variables $R_{ki}(t)$, $R_{ij}(t)$, $R_{iB_{l}}(t)$, and $R_{iW_{l}}(t)$ can be substituted with $R_{ki}[m]$, $R_{ij}[m]$, $R_{iB_{l}}[m]$, and $R_{iW_{l}}[m]$ (m∈ℳ), and each of them remains unchanged within phase m. Therefore, the counterparts of constraints Equations (1), (3)–(5), are as follows:

(10) $$R_{i}+\sum_{k\in {𝒩}_{s},k\neq i}I_{k}R_{ki}[m]=\sum_{j\in {𝒩}_{s},j\neq i}I_{j}R_{ij}[m]+\sum_{B_{l}\in {𝒩}_{B}}I_{iB_{l}}R_{iB_{l}}[m]+\sum_{W_{l}\in {𝒩}_{W}}I_{iW_{l}}R_{iW_{l}}[m]\;(m\in ℳ)$$

(11) $$\begin{array}{l} p_{i}[m]=\sum_{k\in {𝒩}_{s},k\neq i}\rho I_{ik}R_{ki}[m]+\sum_{j\in {𝒩}_{s},j\neq i}C_{ij}I_{ij}R_{ij}[m]+\sum_{B_{l}\in {𝒩}_{B}}C_{iB_{l}}I_{iB_{l}}R_{iB_{l}}[m]+\sum_{W_{l}\in {𝒩}_{W}}C_{iW_{l}}I_{iW_{l}}R_{iW_{l}}[m]\\ \left(m\in ℳ\right) \end{array}$$

(12) $$\sum_{m\in ℳ}\tau_{m}p_{i}[m]=U\tau_{i}$$

(13) $$\tau =\sum_{m\in ℳ}\tau_{m}$$

Combining all the constraints together, we have an optimization problem OPT-3:

$$\begin{array}{l} \text{OPT-3}\\ \max:\frac{\tau_{S}}{\tau }\\ \text{subject to}:(10),(7),(9),(11),(12),(13),(6) \end{array}$$

The optimization variables are now $R_{ki}[m]$, $R_{ij}[m]$, $R_{iB_{l}}[m]$, $R_{iW_{l}}[m]$, P, τ, $\tau_{m}$, $\tau_{S}$, $e_{i}(t)$. In the next section, we will focus on the justification of the equal optimality of OPT-3 and OPT-2.

### 3.3. The Equal Optimality of OPT-3 and OPT-2

**Theorem 1.** *Suppose that* Φ *is the set of all feasible solutions to OPT-2. For arbitrary feasible solution* ϕ∈Φ *with the objective value of OPT-2 known as* $O_{2}(\phi )$*, there must exists a feasible solution to OPT-3, denoted as* $\hat{\phi }$*, which have the same objective value of OPT-2, denoted as* $O_{3}(\hat{\phi })$*, i.e.,* $O_{3}(\hat{\phi })=O_{2}(\phi )$*. What is more, the optimal value of OPT-3 cannot exceed that of OPT-2. Therefore, it can be concluded that OPT-3 and OPT-2 have the equal optimality.*

**Proof.** The similar proof can be found in our earlier work [39]. Here, we postponed the detailed proof in the Appendix section in order to save space.

## 4. The Analysis of OPT-3 and Its Linearization

In the previous section, by Theorem 1, we come to the conclusion that OPT-3 and OPT-2 yield the same optimality. Thus, the problem of solving OPT-2 is equivalent to that of solving OPT-3. Nevertheless, it is still not easy for us to handle the product terms embedding in the constraints of OPT-3. In this section, we will firstly investigate the necessary conditions for obtaining the optimal solution to OPT-3. Then, efforts will be made to turn OPT-3 into a linear programming problem, which is easy to be solved by some already existing tools, such as Lindo, CPLEX, *etc.*

### 4.1. Two Necessary Conditions for the Optimality of OPT-3

**Theorem 2. (The optimal travelling path)** *Suppose that in sub network* $SN_{c}$*, there are* $N_{c}$ *sensor nodes. Then the number of available traveling path among all the sensor nodes and service station* S *is* $N_{c}!$ *(Assume that the wireless energy transfer device always travels along a straight line between two successive spots). The OPT-3 achieves its optimal solution only if the travelling path of the wireless energy transfer device is the shortest Hamilton cycle connecting all sensor nodes and the service station* S*.*

**Proof.** The proof of this theorem is based on the concept of Directed Graph. The full version of this proof is elaborated in the Appendix section.

**Theorem 3. (The existence of “bottleneck” nodes)** *Wireless sensor nodes are defined as “bottleneck” nodes if their remaining battery energy falls exactly to* $E_{min}$ *when the wireless energy-transfer device arrives. Suppose that* $\phi^{⋆}$ *is an optimal solution to OPT-3, then there must exist at least one bottleneck sensor node in the WSN.*

**Proof.** Please refer to the Appendix for more details.

### 4.2. The Linearization of OPT-3

It is still not easy to obtain the optimal solution to OPT-3 since the product terms in the constraints, such as $\tau_{m}p_{i}[m]$, *etc.* In this section, this nonlinear programming problem OPT-3 is turned into a linear programming problem by substituting variables. First, let:

$$\left\{ \begin{array}{l} \eta_{S}=\frac{\tau_{S}}{\tau }\\ \eta_{m}=\frac{\tau_{m}}{\tau }\\ h=\frac{1}{\tau }\\ f_{ij}[m]=R_{ij}[m]\eta_{m}\\ f_{iB_{l}}[m]=R_{iB_{l}}[m]\eta_{m}\\ f_{iW_{l}}[m]=R_{iW_{l}}[m]\eta_{m} \end{array}\right.$$

The objective function of OPT-3 becomes $\eta_{S}$. Constraint Equation (10) is reshaped as:

(14) $$R_{i}\eta_{m}+\sum_{k\in {𝒩}_{s},k\neq i}I_{ki}f_{ki}[m]=\sum_{j\in {𝒩}_{s},j\neq i}I_{ij}f_{ij}[m]+\sum_{B_{l}\in {𝒩}_{B}}I_{iB_{l}}f_{iB_{l}}[m]+\sum_{W_{l}\in {𝒩}_{W}}I_{iW_{l}}f_{iW_{l}}[m]\;(m\in ℳ)$$

The constraint Equations (7) and (9) can be unified as:

(15) $$\eta_{S}\leq \eta_{0}-\frac{\tau_{P}}{E_{\text{max}}-E_{\text{min}}}\left(\sum_{m\in M,m\neq i}\eta_{m}p_{i}[m]\right)\;(m\in ℳ)$$

The constraint Equations (11) and (12) can be unified as:

(16) $$\begin{array}{l} \sum_{m\in M}\left(\sum_{k\in {𝒩}_{s},k\neq i}\rho I_{ki}f_{ki}[m]+\sum_{j\in {𝒩}_{s},j\neq i}C_{ij}I_{ij}f_{ij}[m]+\sum_{B_{l}\in {𝒩}_{B}}C_{iB_{l}}I_{iB_{l}}f_{iB_{l}}[m]+\sum_{W_{l}\in {𝒩}_{W}}C_{iW_{l}}I_{iW_{l}}f_{iW_{l}}[m]\right)=U\eta_{i}\\ \left(m\in ℳ\right) \end{array}$$

The constraint Equation (13) is reformulated as:

(17) $$\sum_{m\in ℳ}\eta_{m}=1$$

The constraint Equation (6) can be omitted here since the optimal travelling path P, which is the shortest Hamilton cycle, is determined in advance. Now, we have the new optimization problem, denoted as OPT-4.

$$\begin{array}{l} \text{OPT-4}\\ \max:\eta_{S}\\ \text{suject to:(14)-(17)} \end{array}$$

The optimization variables are $f_{ij}[m]$, $f_{iB_{l}}[m]$, $f_{iW_{l}}[m]$, $\eta_{m}$, $I_{ij}$, $I_{iB_{l}}$ and $I_{iW_{l}}$. OPT-4 is then a linear optimization problem after determining the values of all indicator functions by calculating the Voronoi diagram of area D.

## 5. The Pre-Normal Replenishing Stage

In last section, two necessary conditions for the optimal solution are given. Additionally, by substituting variables in OPT-3, we obtain OPT-4, and this problem can be simplified into a linear programming problem by determining values of indicator functions in advance. In this section, we will complete our discussion by studying the pre-normal replenishing stage.

The above modeling and corresponding optimization problems are only reasonable for normal replenishing cycles. The energy-time curve of sensor node $s_{i}$ in the pre-normal replenishing stage is not the periodic extension of any curve in normal replenishing cycle since the initial battery energy of arbitrary sensor node in pre-normal replenishing stage is $E_{max}$ , which is higher than the initial battery energy, denoted as $E_{i}$, in any normal replenishing cycle. Nevertheless, as stated in Theorem 4, we can find a solution to the pre-normal replenishing stage by taking advantage of the solution to the normal replenishing cycle, which will make the transition between the pre-normal stage and the first normal cycle smoothly.

**Theorem 4.** *Assume that the length of the pre-normal replenishing stage is* $\tau^{pn}$*, and the time interval of this pre-normal replenishing stage is* $\left[0,\tau^{pn}\right)$*. This time interval is also divided into* $N_{c}+1$ *phases. Denote that the optimal solution to OPT-4 is* ϕ*. The length of the normal replenishing cycle is* τ*, and the data routing scheme associated with phase* m *is* $\left(G_{m}(V,E),\tau_{m}\right)$ *(The meaning of this term is explained in the Appendix). In pre-normal replenishing stage, we have the following equations:*

(18) $$\left\{ \begin{array}{l} \tau^{pn}=\tau \\ (G_{m}^{pn}(V,E),\tau_{m}^{pn})=\left(G_{m}(V,E),\tau_{m}\right)\\ t_{i}^{pn}=t_{i}-\tau^{pn}=t_{i}-\tau \end{array}\right.$$

Denote the wireless energy replenishing power for sensor node $s_{i}$ in the pre-normal stage as $U_{i}^{pn}$. If the power satisfies $U_{i}^{pn}=U-\frac{E_{\text{max}}-E_{i}}{\tau_{i}}$, then:

(I) $e_{i}(t)\geq E_{\text{min}},t\in [0,\tau )$;
(II) $e_{i}(t_{i}^{pn}+\tau_{i}^{pn})=E_{max}$;
(III) $e_{i}(\tau^{pn})=E_{i}$.

**Proof.** Please refer to the Appendix for more details.

## 6. The Simulation and Numerical Analysis

### 6.1. The Simulation Scenarios

Suppose that there are 150 sensor nodes deployed in an area *D*. Additionally, in *D*, we have three base stations. Next to each base station, there is one service station which accommodates one wireless energy transfer device. We make an assumption here that the location information of sensor nodes, base stations and service stations is revealed to us in advance. Assume that the area *D* is a 1000 m × 1000 m square area. The travelling velocity of any wireless energy transfer device is *V* which is set to five meters per second here, and it charges sensor nodes with the power of *U* which is five Watts. The minimum energy required to keep sensor nodes functioning is set to five percent of its maximum energy, *i.e.*, *E_min_* = 0.05*E_max_*. In this paper, *E_max_* is equal to 10,800 J. The sending and receiving energy factors, such as *C_ij_* and *ρ*, are adopted from [40].

### 6.2. Simulation Results and Numerical Analysis

**Figure 6:**
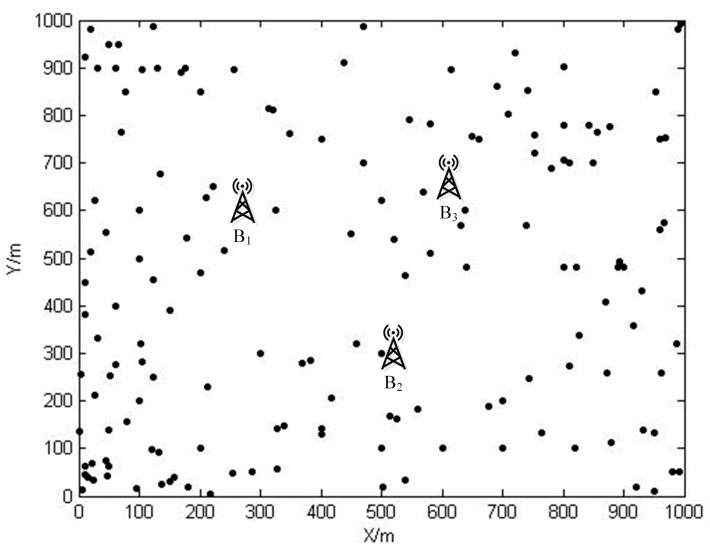
The sensor nodes in area *D*.

**Figure 7:**
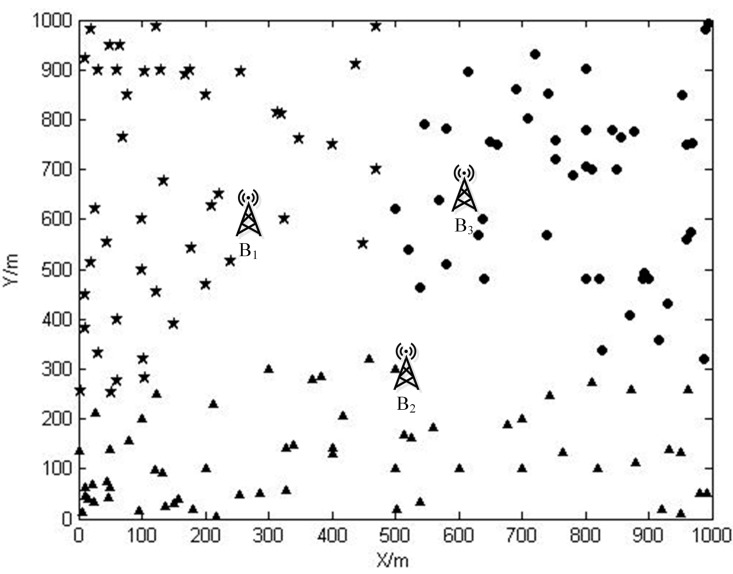
Network partition via Voronoi diagram.

The locations of sensor nodes are shown in Figure 6. The features of sensor nodes are listed in Table 2. In the second column of this table, there are coordinates of sensor nodes, base stations, and service stations. The third column lists the data rate *R_i_* generated by each sensor node. Base stations *B*_1_, *B*_2_ and *B*_3_ are located at (326.8, 800), (500, 300), and (637.2, 600). After calculating the Voronoi diagram, the entire WSN is then partitioned into three sub networks, as shown in Figure 7. Sensor nodes belong to $SN_{1}$, $SN_{2}$, and $SN_{3}$ are represented by pentagram, triangle, and dot symbols, respectively.

**Table 2:** Features of sensor nodes.

Node No.	Coordinates	R_i_(Kbits/s)	Node No.	Coordinates	R_i_(Kbits/s)	Node No.	Coordinates	R_i_(Kbits/s)
1	222 650	12	51	700 200	15	101	213 229	14
2	210 627	14	52	744 247	16	102	300 300	15
3	179 541	15	53	810 274	16	103	370 280	15
4	240 517	15	54	872 259	15	104	383 286	12
5	200 470	15	55	963 258	19	105	459 320	12
6	100 500	15	56	933 139	11	106	740 570	14
7	123 456	16	57	950 132	16	107	800 480	16
8	150 390	16	58	980 50	15	108	822 482	11
9	103 320	11	59	992 50	11	109	871 409	16
10	104 282	19	60	950 10	14	110	827 338	15
11	62 277	19	61	920 20	14	111	915 357	16
12	52 254	12	62	879 114	16	112	987 321	14
13	4 255	14	63	820 100	15	113	929 430	15
14	30 333	12	64	765 133	16	114	890 480	15
15	10 383	15	65	700 100	14	115	900 480	11
16	60 400	19	66	600 100	15	116	894 494	16
17	10 450	16	67	539 34	16	117	960 560	16
18	19 512	11	68	502 19	19	118	967 574	12
19	45 555	11	69	500 100	14	119	850 700	15
20	26 620	11	70	400 130	19	120	802 706	11
21	100 600	15	71	400 141	12	121	810 700	15
22	134 676	12	72	340 149	12	122	780 690	15
23	70 764	14	73	327 142	14	123	754 720	11
24	77 850	12	74	327 57	11	124	752 760	12
25	60 900	16	75	287 50	14	125	800 780	14
26	30 900	14	76	254 47	12	126	843 780	11
27	10 923	16	77	217 5	16	127	856 766	11
28	19 980	15	78	180 19	15	128	877 777	15
29	50 950	14	79	200 100	15	129	960 750	19
30	66 950	11	80	133 93	12	130	970 754	11
31	123 988	19	81	120 98	11	131	953 850	11
32	104 897	14	82	157 39	12	132	990 980	16
33	130 900	16	83	150 30	14	133	995 994	11
34	176 899	12	84	136 24	12	134	802 902	14
35	170 890	15	85	95 15	14	135	720 930	14
36	200 850	19	86	47 41	11	136	614 897	15
37	256 897	16	87	24 33	15	137	690 860	12
38	470 988	15	88	5 14	12	138	741 852	16
39	437 912	19	89	15 39	12	139	710 804	11
40	313 814	12	90	10 45	12	140	650 757	12
41	320 810	14	91	10 63	11	141	660 750	15
42	349 761	16	92	22 68	16	142	580 783	15
43	400 750	16	93	50 62	11	143	545 790	16
44	470 700	14	94	44 75	12	144	568 638	14
45	450 550	14	95	2 137	15	145	500 620	14
46	418 207	19	96	50 140	14	146	520 540	14
47	513 167	12	97	80 157	16	147	539 464	14
48	525 163	14	98	27 213	14	148	580 510	16
49	559 183	15	99	100 200	15	149	640 480	12
50	678 190	12	100	123 250	12	150	630 570	15

**Figure 8:**
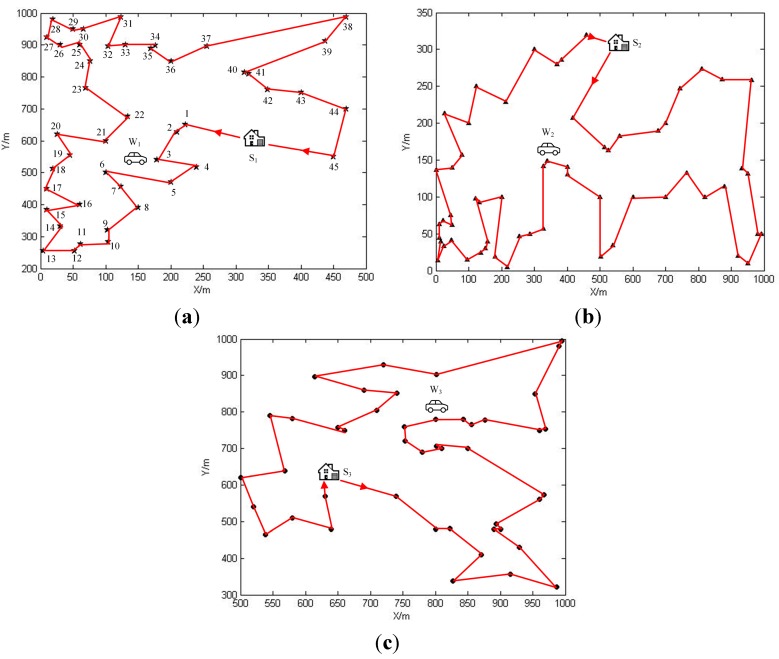
(**a**) The optimal travelling path in $SN_{1}$; (**b**) The optimal travelling path in $SN_{2}$; (**c**) The optimal travelling path in $SN_{3}$.

As stated before, values of indicator functions of OPT-4 will be determined after the network partition is done. The next step is about to solve the optimal traveling path P of the wireless energy transfer device of each sub network. As mentioned in Theorem 2, the optimal travelling path P is the shortest Hamilton cycle connecting all sensor nodes and the service station. This path can be obtained by certain existing tools, such as concorde, which is developed by the Math Department of the University of Waterloo [41]. The optimal path for each sub network is calculated and drawn in Figure 8. In the following discussion, we will intensively investigate the simulation results by taking sub network $SN_{1}$ for example.

**Figure 9:**
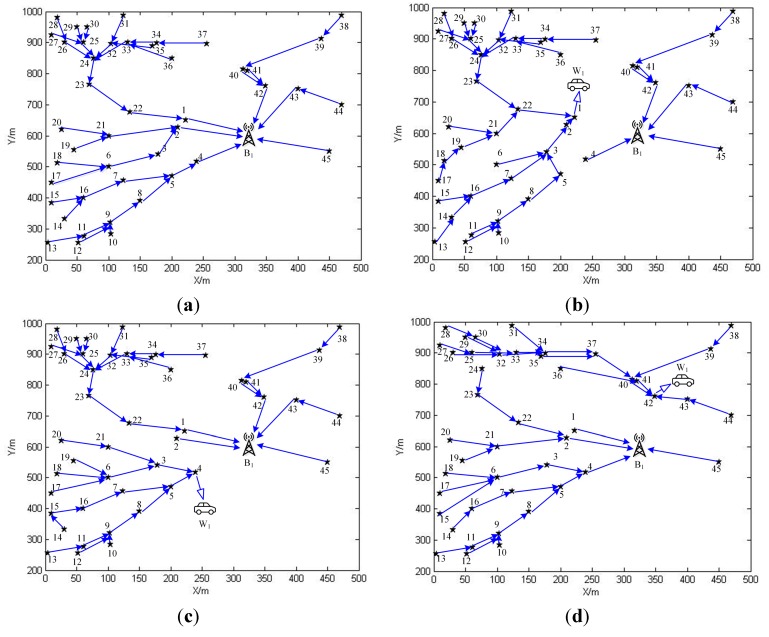
(**a**) The data routing schemes in phase 0 for $SN_{1}$; (**b**) The data routing schemes in phase 1 for $SN_{1}$; (**c**) The data routing schemes in phase 4 for $SN_{1}$; (**d**) The data routing schemes in phase 42 for $SN_{1}$.

**Figure 10:**
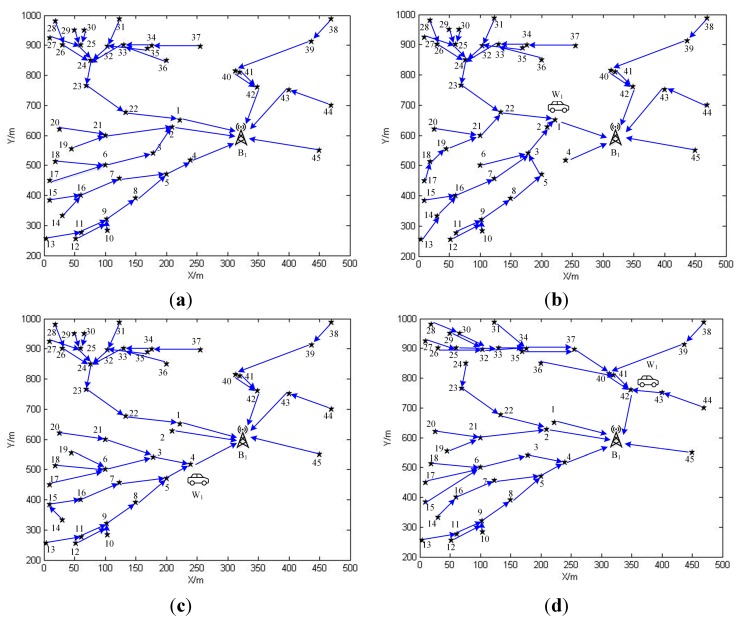
(**a**) The data routing schemes in phase 0 for $SN_{1}$ in the previous work; (**b**) The data routing schemes in phase 1 for $SN_{1}$ in the previous work; (**c**) The data routing schemes in phase 4 for $SN_{1}$ in the previous work; (**d**) The data routing schemes in phase 42 for $SN_{1}$ in the previous work.

In sub network $SN_{1}$, since there are 45 sensor nodes, the normal replenishing cycles could be split into 46 phases. The linear programming problem, OPT-4, can be solved by IBM ILOG Sphere CPLEX or Lindo API. After solving it, we are able to obtain the working strategies for both sensor nodes and the wireless energy transfer device in the form of $\left(G_{m}(V,E),\tau_{m}\right)$ pairs. $G_{m}(V,E)$ with respect to phase m offers the data routing scheme which sensor nodes should adopt in this phase, while $\tau_{m}$ is responsible for the time duration that the wireless energy transfer device should spend on charging and retrieving data from a specific sensor node. It may not be possible for us to draw routing schemes corresponding to all phases due to the limited space, therefore please allow us to pick up certain phases for future discussing. The data routing schemes of phase 0, 1, 4, and 42 are drawn as shown in Figure 9. The reason why these phases are chosen will be elaborated afterwards. The time which is spent by the wireless energy transfer device on staying at service station is 125,347 s, and the sojourn time ratio is about 87.4%. If we apply the working scheme described in the previous work [39] to $SN_{1}$, the optimal sojourn time ration, which can be obtained from solving the formulated optimization problem in the previous work, is about 58.7%. Therefore, the objective value is increased by nearly 50%. The data routing schemes of phase 0, 1, 4, and 42 are drawn as shown in Figure 10. From Figure 10b–d, we can figure out that in phase 1, 4, and 42, sensor nodes 1, 4, and 42 transmit data to the base station over a much longer distance. Hence, the power usages of these sensor nodes are higher than those with respect to the working scheme proposed in this paper, which leads to more energy consumptions. This is the main reason accounting for the relatively smaller sojourn time ratio in the previous work.

**Figure 11:**
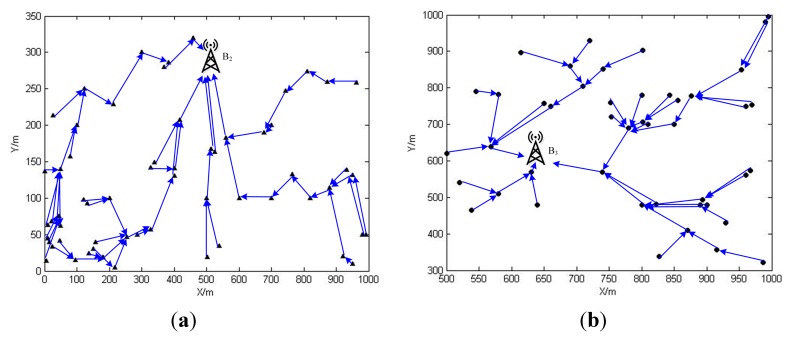
(**a**) The data routing schemes in phase 0 for $SN_{2}$; (**b**) The data routing schemes in phase 0 for $SN_{3}$.

The data routing schemes for $SN_{2}$ and $SN_{3}$ in phase 0 are depicted in Figure 11.

**Table 3:** Results retrieved from the solution to OPT-4 for *SN*_1_.

Node No.	Coordinates	R_i_(Kbits/s)	Arrival Time(s)	Charging Time(s)	Remaining Battery Energy (kJ)
1	222 650	12	143,383.35	2073.44	0.58
2	210 627	14	145,461.98	874.59	6.50
3	179 541	15	146,354.85	252.28	9.65
4	240 517	15	146,620.24	2063.12	0.54
5	200 470	15	148,695.70	535.50	8.67
6	100 500	15	149,252.08	198.43	9.83
7	123 456	16	149,460.44	247.30	9.58
8	150 390	16	149,722.01	498.50	8.31
9	103 320	11	150,237.37	336.27	9.06
10	104 282	19	150,581.24	28.91	10.66
11	62 277	19	150,618.61	81.19	10.37
12	52 254	12	150,704.82	38.97	10.61
13	4 255	14	150,753.39	28.54	10.66
14	30 333	12	150,798.37	34.47	10.65
15	10 383	15	150,843.61	28.89	10.67
16	60 400	19	150,883.06	192.9	9.82
17	10 450	16	151,090.11	91.98	10.34
18	19 512	11	151,194.62	39.55	10.63
19	45 555	11	151,244.22	35.19	10.67
20	26 620	11	151,292.95	30.14	10.66
21	100 600	15	151,338.42	315.94	8.31
22	134 676	12	151,671.01	1230.68	4.14
23	70 764	14	152,923.46	1716.06	2.05
24	77 850	12	154,656.77	990.56	5.83
25	60 900	16	155,657.89	156.12	10.10
26	30 900	14	155,820.01	86.10	10.28
27	10 923	16	155,912.21	28.58	10.66
28	19 980	15	155,952.33	44.98	10.56
29	50 950	14	156,005.94	24.18	10.68
30	66 950	11	156,031.32	20.83	10.71
31	123 988	19	156,067.56	81.21	10.39
32	104 897	14	156,167.36	136.82	10.25
33	130 900	16	156,309.41	231.85	9.65
34	176 899	12	156,550.46	91.06	10.44
35	170 890	15	156,643.69	33.19	10.63
36	200 850	19	156,686.88	68.46	10.47
37	256 897	16	156,769.96	87.65	10.57
38	470 988	15	156,904.12	47.87	10.56
39	437 912	19	156,968.56	857.96	6.53
40	313 814	12	157,858.13	174.13	10.10
41	320 810	14	158,033.26	31.57	10.66
42	349 761	16	158,077.36	2064.68	0.54
43	400 750	16	160,152.47	910.50	6.13
44	470 700	14	161,080.17	49.66	10.56
45	450 550	14	161,160.10	186.72	9.86

In these figures, the solid arrow drawn from one sensor node to the other represents that the former one sends data to the later one. The hollow arrow pointing toward the wireless energy transfer device means the sensor node is sending data directly to the replenishing device while being charged. The number next to each sensor node represents the visiting order of it, *i.e.*, the number “1” means that the sensor node is the first node along the travelling path.

In Table 3, the forth column contains the time at which the wireless energy transfer device arrives at the i-th node along the path P. The fifth column lists the time durations spent on charging different nodes. The sixth column gives the remaining battery energy of each sensor node when visited by the wireless energy transfer device.

The dynamic nature of the data routing schemes: The dynamic nature of the data routing schemes is obvious since $G_{0}(V,E)\neq G_{42}(V,E)$. Specifically, in phase 42, the 42nd sensor node receives data from 41st, 40th and 43rd nodes, and then sends data to $W_{1}$, while in phase 0, it sends data to $B_{1}$, and receives data only from 40th and 41st nodes.

The cyclic nature of the date routing schemes: OPT-4 is based on the modeling of the first normal replenishing cycle. And the optimal solution to it is also applied to the normal replenishing cycles later on, which embodies the cyclic nature. The cyclic nature of the data routing schemes will be fully exhibited by the energy-time curve of a sensor node lately.

The “bottleneck” sensor nodes: There must exist at least one “bottleneck” sensor node according to Theorem 3. Therefore, we will try to locate these sensor nodes and find a way to eliminate these nodes in the sequel. The sensor node $s_{i}$ which is the i-th sensor node along the path P is the bottleneck node if and only if the following equation holds:

(19) $$E_{min}-p_{i}[i]\tau_{i}+U\tau_{i}=E_{max}$$

**Figure 12:**
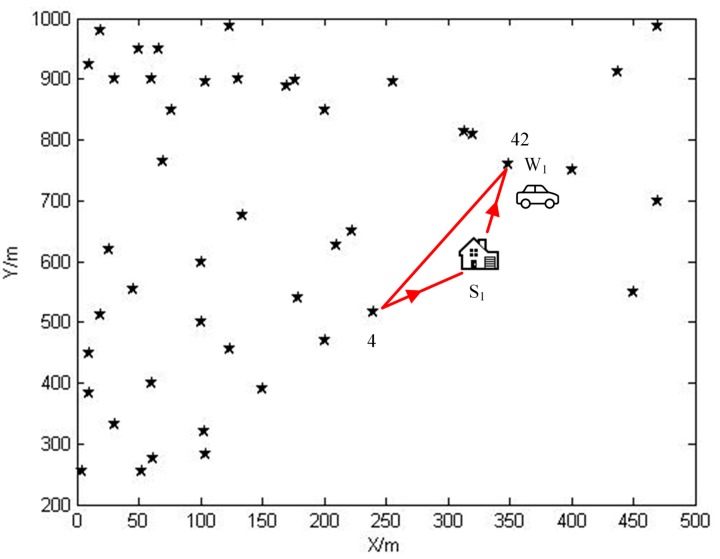
The travelling path for the extra replenishing assignment.

After calculating the power used by all sensor nodes in different phases, we find that the 4th and 42nd along P satisfy Equation (9). Therefore, they are both bottleneck nodes. In addition, it is also the reason why we draw the routing schemes in phase 4 and 42. The existence of bottleneck nodes do harm to the stability of WSNs. Therefore, for this kind of sensor nodes, we developed a “cushion” strategy in case of their energy level falling below $E_{min}$. This strategy is implemented by conducting an extra replenishing assignment during the sojourn time both in pre-normal replenishing stage and normal replenishing cycles only for bottleneck sensor nodes. During the extra replenishing assignment, the wireless energy transfer device visits bottleneck nodes one after another, and offers them with an extra amount of energy, which increases their energy by five percent of their maximum energy. The travelling path is the shortest Hamilton cycle connecting all bottleneck sensor nodes and the service station as shown in Figure 12. The energy replenishing rate applied in the normal replenish assignment is changed to U′ watts. Hence, the initial energy of each normal replenishing cycle will be $E_{i}+0.05E_{max}$ and the minimum remaining energy $e_{i}(t_{i})$ for these nodes will be $E_{min}+0.05E_{max}$. In addition, the replenishing rate for bottleneck sensor node $s_{i}$, which is the i-th sensor node along P, during phase i will be adjusted according to the following equation:

$$E_{min}+0.05E_{max}-p_{i}[i]\tau_{i}+U\prime \tau_{i}=E_{max}$$

thus:

(20) $$U\prime =\frac{0.95E_{max}-E_{min}}{\tau_{i}}+p_{i}[i]=\frac{0.9E_{max}}{\tau_{i}}+p_{i}[i]=U-\frac{0.05E_{max}}{\tau_{i}}$$

The extra replenishing rate of the sensor node 42 in normal replenishing cycles is adjusted to 4.73 W in accordance with Equation (20), and the extra replenishing time duration $\tau_{i}^{ex}$ can be calculated as:

(21) $$\tau_{i}^{ex}=\frac{0.05E_{max}}{U-p_{i}[0]}$$

For the 42nd sensor node, the duration of the extra replenishing operation is about 110 s.

The pre-normal replenishing stage: The working strategies for sensor nodes and the wireless energy transfer device in the pre-normal replenishing stage are almost the same as those in normal replenishing cycles. The energy charging rate is adjusted according to Theorem 4, which is approximately 0.37 W for 42nd sensor node. The power usages of the 42nd sensor node in each phase are listed in Table 4.

**Table 4:** Power usage of the 42nd sensor node in each phase.

Phase No.	Power of 42nd Node (W)	Phase No.	Power of 42nd Node (W)
0	0.0759	23	0.0011
1	0.0759	24	0.0047
2	0.0759	25	0.0047
3	0.0759	26	0.0047
4	0.0759	27	0.0047
5	0.0759	28	0.0047
6	0.0759	29	0.0047
7	0.0759	30	0.0047
8	0.0759	31	0.0047
9	0.0759	32	0.0047
10	0.0759	33	0.0047
11	0.0759	34	0.0047
12	0.0759	35	0.0047
13	0.0759	36	0.0047
14	0.0759	37	0.0047
15	0.0759	38	0.0295
16	0.0759	39	0.0154
17	0.0759	40	0.0047
18	0.0759	41	0.0047
19	0.0759	42	0.0307
20	0.0759	43	0.0267
21	0.0759	44	0.0204
22	0.0154	45	0.0759

**Figure 13:**
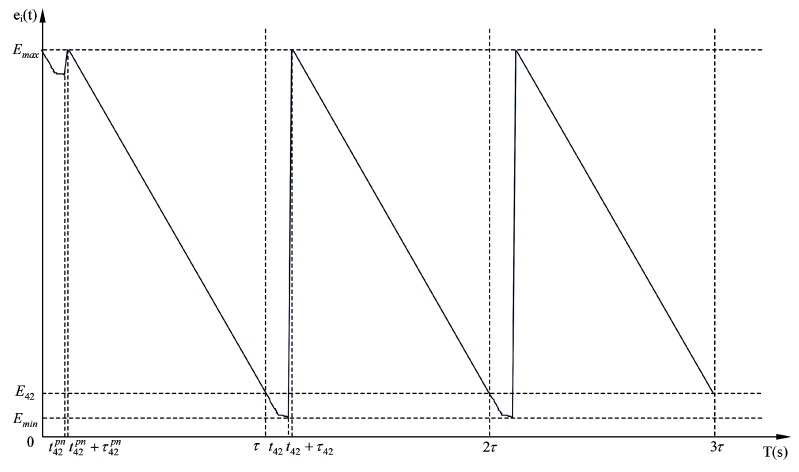
The energy-time curve of the 42nd sensor node without extra energy replenishing in the pre-normal replenishing stage and the 1st and 2nd normal replenishing cycles.

**Figure 14:**
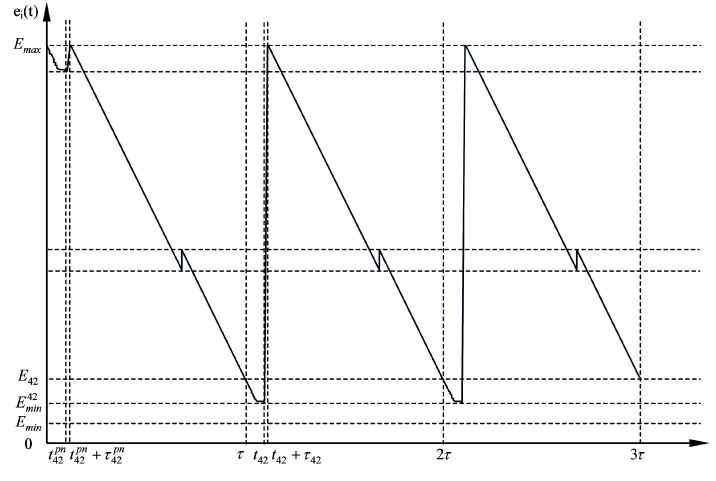
The energy-time curve of the 42nd sensor node with extra energy replenishing in the pre-normal replenishing stage and the 1st and 2nd normal replenishing cycles.

The energy-time curve combining the pre-normal stage, the first normal cycle and the second normal cycle of the 42nd sensor node with and without extra replenishing assignment are drawn in Figure 13 and Figure 14, respectively. From the energy consumption curve over time in the first and second normal cycle, we can figure out the cyclic nature of working strategies of sensor nodes and the wireless energy transfer device.

The extra replenishing in the pre-normal energy replenishing stage starts at 98,013 s, and ends at 98,123 s. The remaining battery energy at the beginning of each normal cycle is 4.68 kJ, and 5.22 kJ when the extra replenishing ends.

The values of notations in Figure 13 and Figure 14 are listed in Table 5.

**Table 5:** Notations and their values in Figure 13 and Figure 14.

Notations	Values
τ	143,360 s
$t_{42}^{pn}$	14,717 s
$t_{42}^{pn}+\tau_{42}$	16,782 s
$t_{42}$	158,077 s
$t_{42}+\tau_{42}$	160,142 s
$E_{max}$	10.80 kJ
$E_{min}$	0.54 kJ
$E_{min}^{42}$	1.08 kJ
$E_{42}$	1.24 kJ (without extra replenishing in Figure 13) 1.78 kJ (with extra replenishing in Figure 14)

## 7. Conclusions

In this article, we investigate the working strategies of sensor nodes and wireless energy transfer devices in WSNs with multiple base stations. The entire sensor network is firstly divided into sub networks according to the concept of Voronoi diagram. The whole energy replenishing procedure is firstly categorized into the pre-normal and normal replenishing stages. By enumerating constraints that sensor nodes and wireless energy transfer devices should comply with, we obtain the optimization problem OPT-1 for the normal replenishing cycles. After dividing the cycle into individual phases, we reach the multi-phased optimization problem OPT-3 which retains the same optimality as OPT-2. OPT-3 is linearized through variable substitution, and then we have OPT-4. Later on, issues relating to the pre-normal replenishing stage are also studied in this article. The simulation conducted in this paper offers us the working strategies of different phases, denoted as $\left(G_{m}(V,E),\tau_{m}\right)$, for both sensor nodes and wireless energy transfer devices. After analyzing the simulation results, the dynamic and cyclic natures of the strategies are revealed. In addition, the bottleneck nodes are pointed out with the help of Equation (19), and an extra replenishing assignment is carried out to provide a cushion for these sensor nodes.

## Figures and Tables

**Figure A1:**
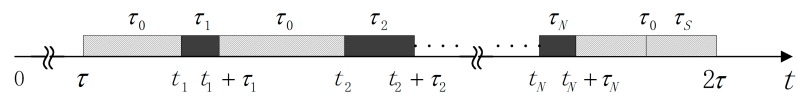
The ordering of $\tau_{m}$ on time axis in the first normal replenishing cycle.

## Appendix

**Proof of Property 1.:**

For the sensor node $s_{i}$, which is the i-th sensor node along the travelling path P, the normal replenishing cycle could be split into three intervals, $\left[\tau ,t_{i}\right)$, $\left[t_{i},t_{i}+\tau_{i}\right)$, and $\left[t_{i}+\tau_{i},2\tau \right)$. In the first interval, since there is no device charging this node, the maximum and minimum remaining battery energy is $e_{i}(\tau )$ and $e_{i}(t_{i})$. In the second interval, the maximum and minimum remaining battery energy is $e_{i}(t_{i}+\tau_{i})$ and $e_{i}(t_{i})$. In the third interval, the maximum and minimum remaining battery energy is $e_{i}(t_{i}+\tau_{i})$ and $e_{i}(2\tau )$. Therefore, the minimum remaining battery energy during the normal replenishing cycle is $\min\{e_{i}(t_{i}),e_{i}(2\tau )\}$. Since we have $e_{i}(2\tau )=e_{i}(\tau )>e_{i}(t_{i})$, the minimum energy will be $e_{i}(t_{i})$. The maximum energy is $\max\{e_{i}(\tau ),e_{i}(t_{i}+\tau_{i})\}$. Since we have $e_{i}(t_{i}+\tau_{i})>e_{i}(2\tau )=e_{i}(\tau )$, the maximum energy remained is $e_{i}(t_{i}+\tau_{i})$. Therefore, the Equations (A.1) and (A.2) hold.

(A.1) $$e_{i}(t_{i})\geq E_{min}$$

(A.2) $$e_{i}(t_{i}+\tau_{i})\leq E_{max}$$

**Proof of Theorem 1.:**

The proof of Theorem 1 can be split into two parts. The proof of the first part is based on construction. Assume that ϕ is a feasible solution to OPT-2, and $\phi =\left\{R_{ki}(t),R_{ij}(t),R_{iB_{l}}(t),R_{iW_{l}}(t),P,\tau_{i},\tau_{P},\tau_{S},e_{i}(t)\right\}$. The $\hat{\phi }$ can be constructed from ϕ as follows:

$$\left\{ \begin{array}{l} {\hat{\tau }}_{i}=\tau_{i}\;(i=1,2,...,N_{c})\\ {\hat{t}}_{i}=t_{i}\;(i=1,2,...,N_{c})\\ {\hat{\tau }}_{0}={\hat{\tau }}_{S}+{\hat{\tau }}_{P}\\ \hat{P}=P\\ \hat{\tau }=\tau \\ {\hat{e}}_{i}({\hat{t}}_{i}+{\hat{\tau }}_{i})=E_{max}\;(i=1,2,...,N_{c})\\ T_{m}={\hat{T}}_{m} \end{array}\right.\text{and}\left\{ \begin{array}{l} {\hat{R}}_{ij}[m]=\frac{\int_{t\in {\hat{T}}_{m}}R_{ij}(t)dt}{{\hat{\tau }}_{m}}\;(i=1,2,...N_{c},j\neq i,m\in ℳ)\\ {\hat{R}}_{iB_{l}}[m]=\frac{\int_{t\in {\hat{T}}_{m}}R_{iB_{l}}(t)dt}{{\hat{\tau }}_{m}}\;(i=1,2,...N_{c},m\in ℳ)\\ {\hat{R}}_{iW_{l}}[m]=\frac{\int_{t\in {\hat{T}}_{m}}R_{iW_{l}}(t)dt}{{\hat{\tau }}_{m}}\;(i=1,2,...N_{c},m\in ℳ)\\ {\hat{p}}_{i}[m]=\frac{\int_{t\in {\hat{T}}_{m}}p_{i}(t)dt}{{\hat{\tau }}_{m}}\;(i=1,2,...N_{c},m\in ℳ) \end{array}\right.$$

It is claimed that $\hat{\phi }$ is a feasible solution to OPT-3, which can be verified by plugging above variables back into constraints of OPT-3 and testing whether they are compatible with them. The validation procedure is listed below:

For constraint Equation (10), we have:

(A.3) $$\begin{array}{l} R_{i}+\sum_{k\in {𝒩}_{s},k\neq i}I_{ki}{\hat{R}}_{ki}[m]=R_{i}+\sum_{k\in {𝒩}_{s},k\neq i}\frac{\int_{t\in {\hat{T}}_{m}}I_{ki}R_{ki}(t)dt}{{\hat{\tau }}_{m}}=\frac{\int_{t\in {\hat{T}}_{m}}\left(R_{i}+\sum_{k\in {𝒩}_{s},k\neq i}I_{ki}R_{ki}(t)\right)dt}{{\hat{\tau }}_{m}}\\ =\frac{\int_{t\in {\hat{T}}_{m}}\left(\sum_{j\in {𝒩}_{s},j\neq i}I_{ij}R_{ij}(t)+\sum_{B_{l}\in {𝒩}_{B}}I_{iB_{k}}R_{iB_{l}}(t)+\sum_{W_{l}\in {𝒩}_{W}}I_{iW_{l}}R_{iW_{l}}(t)\right)dt}{{\hat{\tau }}_{m}}\\ =\sum_{j\in {𝒩}_{s},j\neq i}I_{ij}{\hat{R}}_{ij}[m]+\sum_{B_{l}\in {𝒩}_{B}}I_{iB_{l}}{\hat{R}}_{iB_{l}}[m]+\sum_{W_{l}\in {𝒩}_{W}}I_{iW_{l}}{\hat{R}}_{iW_{l}}[m] \end{array}$$

The third equation holds since $R_{ki}(t)$, $R_{ij}(t)$, $R_{iB_{l}}(t)$, and $R_{iW_{l}}(t)$ are elements of the feasible solution ϕ of OPT-2.

For constraint Equation (7), we have:

(A.4) $$\begin{array}{l} {\hat{e}}_{i}({\hat{t}}_{i})={\hat{e}}_{i}({\hat{t}}_{i}+{\hat{\tau }}_{i})-\sum_{m\neq i}{\hat{p}}_{i}[m]{\hat{\tau }}_{m}=E_{max}-\sum_{m\neq i}\frac{\int_{t\in {\hat{T}}_{m}}p_{i}(t)dt}{{\hat{\tau }}_{m}}{\hat{\tau }}_{m}\\ =E_{max}-\sum_{m\neq i}\int_{t\in {\hat{T}}_{m}}p_{i}(t)dt=E_{max}-\left(\int_{t={\hat{t}}_{i}+{\hat{\tau }}_{i}}^{2\hat{\tau }}p_{i}(t)dt+\int_{t=\hat{\tau }}^{{\hat{t}}_{i}}p_{i}(t)dt\right)\\ =e_{i}(t_{i}+\tau_{i})-\left(\int_{t=t_{i}+\tau_{i}}^{2\tau }p_{i}(t)dt+\int_{t=\tau }^{t_{i}}p_{i}(t)dt\right)=e_{i}(t_{i})\geq E_{min} \end{array}$$

For constraint Equation (11), we have:

(A.5) $$\begin{array}{l} {\hat{p}}_{i}[m]=\frac{\int_{t\in {\hat{T}}_{m}}p_{i}(t)dt}{{\hat{\tau }}_{m}}=\frac{\int_{t\in {\hat{T}}_{m}}\left(\sum_{k\in {𝒩}_{s},k\neq i}\rho I_{ki}R_{ki}(t)+\sum_{j\in {𝒩}_{s},j\neq i}C_{ij}I_{ij}R_{ij}(t)+\sum_{B_{l}\in {𝒩}_{B}}C_{iB_{l}}I_{B_{l}}R_{iB_{l}}(t)+\sum_{W_{l}\in {𝒩}_{W}}C_{iW_{l}}I_{W_{l}}R_{iW_{l}}(t)\right)dt}{{\hat{\tau }}_{m}}\\ =\sum_{k\in {𝒩}_{s},k\neq i}\rho \frac{\int_{t\in {\hat{T}}_{m}}I_{ki}R_{ki}(t)dt}{{\hat{\tau }}_{m}}+\sum_{j\in {𝒩}_{s},j\neq i}C_{ij}\frac{\int_{t\in {\hat{T}}_{m}}I_{ij}R_{ij}(t)dt}{{\hat{\tau }}_{m}}+\sum_{B_{l}\in {𝒩}_{B}}C_{iB_{l}}\frac{\int_{t\in {\hat{T}}_{m}}I_{B_{l}}R_{iB_{l}}(t)dt}{{\hat{\tau }}_{m}}+\sum_{W_{l}\in {𝒩}_{W}}C_{iW_{l}}\frac{\int_{t\in {\hat{T}}_{m}}I_{W_{l}}R_{iW_{l}}(t)dt}{{\hat{\tau }}_{m}}\\ =\sum_{k\in {𝒩}_{s},k\neq i}\rho I_{ki}{\hat{R}}_{ki}[m]+\sum_{j\in {𝒩}_{s},j\neq i}C_{ij}I_{ij}{\hat{R}}_{ij}[m]+\sum_{B_{l}\in {𝒩}_{B}}C_{iB_{l}}I_{iB_{l}}{\hat{R}}_{iB_{l}}[m]+\sum_{W_{l}\in {𝒩}_{W}}C_{iW_{l}}I_{iW_{l}}{\hat{R}}_{iW_{l}}[m] \end{array}$$

The second equation holds in that ϕ is a feasible solution to OPT-2.

For constraint Equation (12), we have:

(A.6) $$\begin{array}{l} \sum_{m\in ℳ}{\hat{\tau }}_{m}{\hat{p}}_{i}[m]=\sum_{m\in ℳ}{\hat{\tau }}_{m}\frac{\int_{t\in {\hat{T}}_{m}}p_{i}(t)dt}{{\hat{\tau }}_{m}}=\sum_{m\in ℳ}\int_{t\in {\hat{T}}_{m}}p_{i}(t)dt\\ =\int_{t=\hat{\tau }}^{2\hat{\tau }}p_{i}(t)dt=\int_{t=\tau }^{2\tau }p_{i}(t)dt=U\tau_{i}=U{\hat{\tau }}_{i} \end{array}$$

Constraint Equation (13) holds since

(A.7) $$\hat{\tau }={\hat{\tau }}_{S}+{\hat{\tau }}_{P}+\sum_{i=1}^{N_{c}}{\hat{\tau }}_{i}={\hat{\tau }}_{0}+\sum_{i=1}^{N_{c}}{\hat{\tau }}_{i}=\sum_{m\in ℳ}{\hat{\tau }}_{m}$$

and constraint Equation (6) holds since $\hat{P}=P$.

Therefore, $\hat{\phi }$ is indeed a feasible solution to OPT-3. The objective value achieved by $\hat{\phi }$ can be calculated as:

(A.8) $$O_{3}(\hat{\phi })=\frac{{\hat{\tau }}_{S}}{\hat{\tau }}=\frac{\hat{\tau }-{\hat{\tau }}_{P}-\sum_{i=1}^{N_{c}}{\hat{\tau }}_{i}}{\tau }=\frac{\tau -\tau_{P}-\sum_{i=1}^{N_{c}}\tau_{i}}{\tau }=\frac{\tau_{S}}{\tau }=O_{2}(\phi )$$

*i.e.*, $\hat{\phi }$ yields the same objective value as ϕ does.

The proof of the second half of this theorem is straightforward. Since the values of optimization variables are fixed in phase m∈ℳ, any solution to OPT-3 is also a feasible solution to OPT-2 with less flexibility. Therefore, the optimal solution to OPT-3 is of course a feasible to OPT-2, which means that the optimal value of OPT-3 will not exceed that of OPT-2. In conclusion, OPT-3 and OPT-2 are of the same optimality.

**Proof of Theorem 2. (The Optimal Travelling Path)**

Intuitively, if a wireless energy transfer device spends less time on roaming, it will be able to enjoy more time staying at the service station. The formal proof of this assertion is based on contradiction. Before going through the proof, please allow us to explain some notations used in the proof. Let P denote the travelling path, and P can be represented by $\left\{\pi_{0},\pi_{1},...,\pi_{N_{c}},\pi_{0}\right\}$, where $\pi_{0}$ denotes the service station, and $\pi_{i}(i\neq 0)$ denotes the i-th sensor node along the path P. The phase m(m≠0) commences at the time when the wireless energy transfer device arrives at the m-th sensor node along the travelling path P, and ends at the time when it leaves this node. The ordering of time intervals according to different phases can be interpreted on time axis as shown in Figure A1. The data routing information of this WSN in phase m can be represented by a directed graph $G_{m}(V,E)$, where V stands for the set of all sensor nodes, and E contains the data routing information. The time duration of phase m is denoted as $\tau_{m}$. Suppose that during phase m, the wireless energy transfer device charges the sensor node $s_{i}$, then we can draw a one to one projection from $s_{i}$ to phase m, denoted as $s_{i}↔\left(G_{m}(V,E),\tau_{m}\right)$.

Suppose that $\phi^{⋆}$ is the optimal solution to OPT-3, and that the travelling path $P^{⋆}$ is not the shortest Hamilton cycle connecting all sensor nodes and the service station. We can construct a feasible solution $\hat{\phi }$ from $\phi^{⋆}$, and $O_{3}(\hat{\phi })$ exceeds $O_{3}(\phi^{⋆})$, which yields the contradiction. $\hat{\phi }$ is constructed as follows:

In $\hat{\phi }$, the travelling path $\hat{P}$ is the shortest Hamilton cycle, which can be expressed as $\left\{\pi_{0},{\hat{\pi }}_{1},...,{\hat{\pi }}_{N_{c}},\pi_{0}\right\}$, while the optimal path $P^{⋆}$ in $\phi^{⋆}$ can be represented by $\left\{\pi_{0},\pi_{1}^{⋆},...,\pi_{N_{c}}^{⋆},\pi_{0}\right\}$. Without loss of generality, suppose that, in $\phi^{⋆}$, the sensor node $s_{i}$ is charged by the wireless energy transfer device in phase m, *i.e.*, it is the m-th sensor node along the travelling path $P^{⋆}$. Thus, we have the one-to-one projection $s_{i}↔\left(G_{m}^{⋆}(V,E),\tau_{m}^{⋆}\right)$. In $\hat{\phi }$, the projection will be $s_{i}↔\left({\hat{G}}_{n}(V,E),{\hat{\tau }}_{n}\right)$, which means that the same sensor node $s_{i}$ is the n-th node visited by the wireless energy transfer device and is charged in phase n. Now, let $\left({\hat{G}}_{n}(V,E),{\hat{\tau }}_{n}\right)$ be equal to $\left(G_{m}^{⋆}(V,E),\tau_{m}^{⋆}\right)$, which indicates that no matter during which time span $s_{i}$ is charged, the length of charging time and the data routing graph attached to it remain unchanged. In another word, the ordered set $\left\{\left({\hat{G}}_{1}(V,E),{\hat{\tau }}_{1}\right),\left({\hat{G}}_{2}(V,E),{\hat{\tau }}_{2}\right),...,\left({\hat{G}}_{N_{c}}(V,E),{\hat{\tau }}_{N_{c}}\right)\right\}$ is nothing but a permutation of $\left\{\left(G_{1}^{⋆}(V,E),\tau_{1}^{⋆}\right),\left(G_{2}^{⋆}(V,E),\tau_{2}^{⋆}\right),...,\left(G_{N_{c}}^{⋆}(V,E),\tau_{N_{c}}^{⋆}\right)\right\}$. Now, let ${\hat{\tau }}_{0}=\tau_{0}^{⋆}$, then we have $\hat{\tau }=\tau^{⋆}$. Additionally, let $e_{i}({\hat{t}}_{i}+{\hat{\tau }}_{i})=E_{max}$. Next, we will show that $\hat{\phi }$ constructed is a feasible solution to OPT-3 by checking whether it complies with all constraints of OPT-3.

For constraint Equation (10), we have:

(A.9) $$\begin{array}{l} R_{i}+\sum_{k\in {𝒩}_{s},k\neq i}I_{ki}{\hat{R}}_{ki}[m]=R_{i}+\sum_{k\in {𝒩}_{s},k\neq i}I_{ki}R_{ki}^{⋆}[n]\\ =\sum_{j\in {𝒩}_{s},j\neq i}I_{ij}R_{ij}^{⋆}[n]+\sum_{B_{l}\in {𝒩}_{B}}I_{iB_{l}}R_{iB_{l}}^{⋆}[n]+\sum_{W_{l}\in {𝒩}_{W}}I_{iW_{l}}R_{iW_{l}}^{⋆}[n]\\ =\sum_{j\in {𝒩}_{s},j\neq i}I_{ij}{\hat{R}}_{ij}[m]+\sum_{B_{l}\in {𝒩}_{B}}I_{iB_{l}}{\hat{R}}_{iB_{l}}[m]+\sum_{W_{l}\in {𝒩}_{W}}I_{iW_{l}}{\hat{R}}_{iW_{l}}[m] \end{array}$$

The first and third equations hold in that $\left({\hat{G}}_{n}(V,E),{\hat{\tau }}_{n}\right)=\left(G_{m}^{⋆}(V,E),\tau_{m}^{⋆}\right)$.

Constraint Equation (11) holds for the same reason.

For constraint Equation (7), we have:

(A.10) $$\begin{array}{l} {\hat{e}}_{i}({\hat{t}}_{i})={\hat{e}}_{i}({\hat{t}}_{i}+{\hat{\tau }}_{i})-\sum_{k\neq n}{\hat{p}}_{i}[k]{\hat{\tau }}_{k}=E_{max}-\sum_{k\neq m}p_{i}^{⋆}[k]\tau_{k}^{⋆}\\ =e_{i}(t_{i}+\tau_{i})-\sum_{k\neq m}p_{i}^{⋆}[k]\tau_{k}^{⋆}=e_{i}(t_{i})\geq E_{min} \end{array}$$

The second equation holds since $\left\{\left({\hat{G}}_{1}(V,E),{\hat{\tau }}_{1}\right),\left({\hat{G}}_{2}(V,E),{\hat{\tau }}_{2}\right),...,\left({\hat{G}}_{N_{c}}(V,E),{\hat{\tau }}_{N_{c}}\right)\right\}$ is a permutation of $\left\{\left(G_{1}^{⋆}(V,E),\tau_{1}^{⋆}\right),\left(G_{2}^{⋆}(V,E),\tau_{2}^{⋆}\right),...,\left(G_{N_{c}}^{⋆}(V,E),\tau_{N_{c}}^{⋆}\right)\right\}$.

For constraint Equation (12), we have:

(A.11) $$\sum_{k\in ℳ}{\hat{\tau }}_{k}{\hat{p}}_{i}[k]=\sum_{l\in ℳ}\tau_{l}^{⋆}p_{i}^{⋆}[l]=U\tau_{m}^{⋆}=U{\hat{\tau }}_{n}$$

For constraint Equation (13), we have:

(A.12) $$\hat{\tau }={\hat{\tau }}_{0}+\sum_{i=1}^{N_{c}}{\hat{\tau }}_{i}=\sum_{m\in ℳ}{\hat{\tau }}_{m}$$

Therefore, $\hat{\phi }$ is indeed a feasible solution to OPT-3. The objective value yielded by $\hat{\phi }$ can be calculated as follows:

(A.13) $$O_{3}(\hat{\phi })=\frac{{\hat{\tau }}_{S}}{\hat{\tau }}=\frac{{\hat{\tau }}_{0}-{\hat{\tau }}_{\hat{P}}}{\hat{\tau }}>\frac{{\hat{\tau }}_{0}-\tau_{P^{⋆}}^{⋆}}{\hat{\tau }}=\frac{\tau_{0}^{⋆}-\tau_{P^{⋆}}^{⋆}}{\tau^{⋆}}=\frac{\tau_{S}^{⋆}}{\tau^{⋆}}=O_{3}(\phi^{⋆})$$

The “>” holds since $\hat{P}$ is the shortest Hamilton cycle along which it is less time consumed. Therefore, $\hat{\phi }$ yields better objective value of OPT-3 than $\phi^{⋆}$ does, which contradicts the assumption that $\phi^{⋆}$ is the optimal solution to OPT-3. The proof is complete.

**Proof of Theorem 3. (The Existence of “Bottleneck” Nodes)**

The proof of this theorem is also by contradiction. Suppose that $\phi^{⋆}$ is the optimal solution to OPT-3. What is more, under this solution, there are no bottleneck nodes existing in the WSN, *i.e.*, the minimum remaining energy of all sensor nodes are all greater than $E_{min}$. Denote the minimum remaining energy of sensor node $s_{i}$ as $E_{min}^{i}$, and let the scaling factor be $\gamma =\frac{E_{max}-E_{min}}{\max_{i}\{E_{max}-E_{\text{min}}^{i}\}}$. Evidently, we have γ>1 . By letting:

(A.14) $$\left\{ \begin{array}{l} \hat{P}=P^{⋆}\\ \hat{\tau }=\gamma \tau^{⋆}\\ {\hat{\tau }}_{i}=\gamma \tau_{i}^{⋆}\text{ (}i=1,2,...,N_{k}\text{)}\\ {\hat{R}}_{ij}[m]=R_{ij}^{⋆}[m]\text{ (}m\in ℳ\text{)}\\ {\hat{R}}_{iB_{l}}[m]=R_{iB_{l}}^{⋆}[m]\text{ (}m\in ℳ\text{)}\\ {\hat{R}}_{iW_{l}}[m]=R_{iW_{l}}^{⋆}[m]\text{ (}m\in ℳ\text{)}\\ {\text{e}}_{i}({\hat{t}}_{i}+{\hat{\tau }}_{i})=E_{max} \end{array}\right.$$

we have a candidate solution to OPT-3, denoted as $\hat{\phi }$. What we should do next is to verify the feasibility of $\hat{\phi }$. Firstly, we will show that ${\hat{\tau }}_{0}$ is greater than 0.

(A.15) $${\hat{\tau }}_{0}=\hat{\tau }-\sum_{i=1}^{N_{c}}{\hat{\tau }}_{i}=\gamma \left(\tau^{⋆}-\sum_{i=1}^{N_{c}}\tau_{i}^{⋆}\right)=\gamma {\hat{\tau }}_{0}>0$$

With respect to definitions of ${\hat{R}}_{ij}[m]$, ${\hat{R}}_{iB_{l}}[m]$, ${\hat{R}}_{iW_{l}}[m]$ and $\hat{P}$, we have ${\hat{p}}_{i}[m]=p_{i}^{⋆}[m]$, and ${\hat{\tau }}_{P}=\tau_{P}^{⋆}$. Constraint Equation (10) holds since:

(A.16) $$\begin{array}{l} R_{i}+\sum_{k\in {𝒩}_{s},k\neq i}I_{ki}{\hat{R}}_{ki}[m]=R_{i}+\sum_{k\in {𝒩}_{s},k\neq i}I_{ki}R_{ki}^{⋆}[m]\\ =\sum_{j\in {𝒩}_{s},j\neq i}I_{ij}R_{ij}^{⋆}[m]+\sum_{B_{l}\in {𝒩}_{B}}I_{iB_{l}}R_{iB_{l}}^{⋆}[m]+\sum_{W_{l}\in {𝒩}_{W}}I_{iW_{l}}R_{iW_{l}}^{⋆}[m]\\ =\sum_{j\in {𝒩}_{s},j\neq i}I_{ij}{\hat{R}}_{ij}[m]+\sum_{B_{l}\in {𝒩}_{B}}I_{iB_{l}}{\hat{R}}_{iB_{l}}[m]+\sum_{W_{l}\in {𝒩}_{W}}I_{iW_{l}}{\hat{R}}_{iW_{l}}[m] \end{array}$$

Similarly, constraint Equation (11) holds for the same reason.

For constraint Equation (7), we have:

(A.17) $$\begin{array}{l} e_{i}({\hat{t}}_{i})=e_{i}({\hat{t}}_{i}+{\hat{\tau }}_{i})-\sum_{m\in ℳ,m\neq i}{\hat{p}}_{i}[m]{\hat{\tau }}_{m}\\ =E_{max}-(U{\hat{\tau }}_{i}-{\hat{p}}_{i}[i]{\hat{\tau }}_{i})=E_{max}-\gamma \left(U\tau_{i}^{⋆}-p_{i}^{⋆}[i]\tau_{i}^{⋆}\right)\\ =E_{max}-\frac{E_{max}-E_{min}}{\max_{i}\{E_{max}-E_{\text{min}}^{i}\}}\left(U\tau_{i}^{⋆}-p_{i}^{⋆}[i]\tau_{i}^{⋆}\right)\\ =E_{max}-\frac{E_{max}-E_{min}}{\max_{i}\{e_{i}(t_{i}^{⋆}+\tau_{i}^{⋆})-e_{i}(t_{i}^{⋆})\}}\left(e_{i}(t_{i}^{⋆}+\tau_{i}^{⋆})-e_{i}(t_{i}^{⋆})\right)\\ \geq E_{max}-(E_{max}-E_{min})\\ =E_{min} \end{array}$$

The fifth equation holds since the Property 1 holds and $e_{i}(t_{i}^{⋆})=e_{i}(t_{i}^{⋆}+\tau_{i}^{⋆})-\left(U\tau_{i}^{⋆}-p_{i}^{⋆}[i]\tau_{i}^{⋆}\right)$.

For constraint Equation (12), we have:

(A.18) $$\sum_{m\in ℳ}{\hat{\tau }}_{m}{\hat{p}}_{i}[m]=\sum_{m\in ℳ}\gamma \tau_{m}^{⋆}p_{i}^{⋆}[m]=\gamma U\tau_{i}^{⋆}=U{\hat{\tau }}_{i}$$

Constraint Equation (13) holds in that:

(A.19) $$\hat{\tau }={\hat{\tau }}_{S}+{\hat{\tau }}_{P}+\sum_{i=1}^{N_{c}}{\hat{\tau }}_{i}={\hat{\tau }}_{0}+\sum_{i=1}^{N_{c}}{\hat{\tau }}_{i}=\sum_{m\in ℳ}{\hat{\tau }}_{m}$$

Therefore, $\hat{\phi }$ is a feasible solution to OPT-3. The objective value achieved by $\hat{\phi }$ can be calculated as follow:

(A.20) $$O_{3}(\hat{\phi })=\frac{{\hat{\tau }}_{S}}{\hat{\tau }}=\frac{{\hat{\tau }}_{0}-{\hat{\tau }}_{P}}{\hat{\tau }}=\frac{\gamma \tau_{0}^{\text{⋆}}-\tau_{P}^{\text{⋆}}}{\gamma \tau^{\text{⋆}}}>\frac{\gamma \left(\tau_{0}^{\text{⋆}}-\tau_{P}^{\text{⋆}}\right)}{\gamma \tau^{\text{⋆}}}=\frac{\tau_{S}^{\text{⋆}}}{\tau^{\text{⋆}}}=O_{3}(\phi^{\text{⋆}})$$

The “>” is tenable since γ>1. From the inequality, we can draw the conclusion that $\hat{\phi }$ yields better objective value than $\phi^{⋆}$ does, which contradicts the assumption that $\phi^{⋆}$ is an optimal solution. Hence, there must be at least one bottleneck sensor node in the WSN under optimal solution $\phi^{⋆}$.

**Proof of Theorem 4.**

(A.21) $$\left\{ \begin{array}{l} \tau^{pn}=\tau \\ (G_{m}^{pn}(V,E),\tau_{m}^{pn})=\left(G_{m}(V,E),\tau_{m}\right)\\ t_{i}^{pn}=t_{i}-\tau^{pn}=t_{i}-\tau \end{array}\right.$$

Denote the wireless energy replenishing power for sensor node $s_{i}$ in the pre-normal stage as $U_{i}^{pn}$. If the power satisfies $U_{i}^{pn}=U-\frac{E_{\text{max}}-E_{i}}{\tau_{i}}$, then:

(I) $e_{i}(t)\geq E_{\text{min}},t\in [0,\tau )$;
(II) $e_{i}(t_{i}^{pn}+\tau_{i}^{pn})=E_{max}$;
(III) $e_{i}(\tau^{pn})=E_{i}$.

**Proof.** According to Equation (A.21), we have $e_{i}(t_{i}^{pn})=E_{max}-\left(E_{i}-e_{i}(t_{i})\right)=e_{i}(t_{i})+\left(E_{max}-E_{i}\right)>e_{i}(t_{i})\geq E_{min}$. Similar to Property 1, at $t_{i}^{pn}$, the remaining battery energy falls to its minimum point. Therefore,

(I) holds.
(II) holds since:
  (A.22) $$\begin{array}{l} e_{i}(t_{i}^{pn}+\tau_{i}^{pn})=e_{i}(t_{i}^{pn})-p_{i}^{pn}[i]\tau_{i}^{pn}+U_{i}^{pn}\tau_{i}^{pn}\\ =e_{i}(t_{i})+\left(E_{max}-E_{i}\right)-p_{i}[i]\tau_{i}+\left(U-\frac{E_{\text{max}}-E_{i}}{\tau_{i}}\right)\tau_{i}\\ =e_{i}(t_{i})+U\tau_{i}-p_{i}[i]\tau_{i}=E_{max} \end{array}$$
(III) holds since:
  (A.23) $$\begin{array}{l} e_{i}(\tau^{pn})=e_{i}(t_{i}^{pn}+\tau_{i}^{pn})-\left(e_{i}(t_{i}+\tau_{i})-e_{i}(2\tau )\right)=E_{max}-\left(E_{max}-e_{i}(2\tau )\right)\\ =e_{i}(2\tau )=e_{i}(\tau )=E_{i} \end{array}$$

In conclusion, in the pre-normal replenishing stage, each sensor node and the wireless energy transfer device adopt the same working strategies as those in normal replenishing cycles, and the wireless energy transfer power for sensor node $s_{i}$, which is the i-th sensor node along the travelling path, will be adjusted to $U_{i}^{pn}=U-\frac{E_{\text{max}}-E_{i}}{\tau_{i}}$.
